# Comprehensive characterization of the OCT1 phenylalanine-244-alanine substitution reveals highly substrate-dependent effects on transporter function

**DOI:** 10.1016/j.jbc.2024.107835

**Published:** 2024-09-27

**Authors:** Carla Isabel Wittern, Sophie Schröder, Ole Jensen, Jürgen Brockmöller, Lukas Gebauer

**Affiliations:** 1 Institute of Clinical Pharmacology, University Medical Center Göttingen, Göttingen, Germany; 2 Department for Epigenetics and Systems Medicine in Neurodegenerative Diseases, German Center for Neurodegenerative Diseases (DZNE), Göttingen, Germany

**Keywords:** site-directed mutagenesis, organic cation transporter 1, polyspecificity, transport, inhibition, alanine mutagenesis, binding pocket

## Abstract

Organic cation transporters (OCTs) can transport structurally highly diverse substrates. The molecular basis of this extensive polyspecificity has been further elucidated by cryo-EM. Apparently, in addition to negatively charged amino acids, aromatic residues may contribute to substrate binding and substrate selectivity. In this study, we provide a comprehensive characterization of phenylalanine 244 in OCT1 function. We analyzed the uptake of 144 OCT1 substrates for the phenylalanine 244 to alanine substitution compared to *WT*OCT1. This substitution had highly substrate-specific effects ranging from transport reduced to 10% of WT activity up to 8-fold increased transport rates. Four percent of substrates showed strongly increased uptake (>200% of WT) whereas 39% showed strongly reduced transport (<50% of WT). Particularly with larger, more hydrophobic, and more aromatic substrates, the Phe244Ala substitution resulted in higher transport rates and lower inhibition of the transporter. In contrast, substrates with a lower molecular weight and less aromatic rings showed generally decreased uptake rates. A comparison of our data to available transport kinetic data demonstrates that generally, high-affinity low-capacity substrates show increased uptake by the Phe244Ala substitution, whereas low-affinity high-capacity substrates are characterized by reduced transport rates. Altogether, our study provides the first comprehensive characterization of the functional role of an aromatic amino acid within the substrate translocation pathway of OCT1. The pleiotropic function further highlights that phenylalanine 244 interacts in a highly specific manner with OCT1 substrates and inhibitors.

Membrane transporter proteins of the solute carrier (SLC) family are crucial for the tissue distribution of numerous drugs but also the homeostasis of various endogenous substances (1). The organic cation transporter 1 (OCT1/*SLC22A1*) is highly expressed at sinusoidal membranes of hepatocytes (2) and its clinical relevance for drug pharmacokinetics has been illustrated for several relevant therapeutics (3, 4, 5).

An outstanding feature of OCT1 is its polyspecificity (6, 7) which is illustrated by at least 150 substrates transported *via* OCT1. This is also the case for its related homolog OCT2 and OCT-3 and stands in contrast to other classes of cation membrane transporters such as the high-affinity monoamine transporters which are characterized by a much narrower substrate spectrum (8). OCT substrates are usually, but not exclusively (9), characterized by a positive charge at physiologic pH, a molecular weight between 150 and 450 Da and hydrophilic characteristics. Besides this, OCT substrates are highly diverse in their structure.

The molecular basis of OCT polyspecificity is not well understood although recently published cryo-EM structures of OCTs accelerate the molecular understanding of OCT ligand interactions (10, 11, 12, 13). Ahead of the cryo-EM era, mechanistic studies were mainly based on mutagenesis studies often guided by homology modeling (14). From these studies it was already early concluded that Asp474 in OCT1 is crucial for the electrostatic interaction with the positively charged substrates. However, also the transport uncharged substrates of OCT1 significantly depends on Asp474 (9) which is consistent with recent cryo-EM studies revealing that Asp474 may be more important for stabilization of the OCT1 structure rather than specific ligand interactions (10, 12). Ligand interactions are mainly mediated by Glu386 which was shown to be important for substrate binding (10). This was also shown for the corresponding amino acids in OCT3 (15).

In addition to anionic amino acids, aromatic amino acids in the substrate translocation path may determine substrate selectivity by hydrophobic aromatic stacking interactions with OCT substrates since not all, but most OCT substrates have at least one aromatic ring. In a systematic analysis of all possible OCT1 mutations, aromatic residues were identified as important to coordinate the substrate passage through OCT1 (16). Any mutations to nonaromatic amino acids resulted in a loss of transport function. Among the aromatic residues, phenylalanine at codon 244 was shown to be involved in OCT1 substrate translocation by several independent studies. A prominent role was proposed by *in silico* prediction studies (17, 18), but also by experimental, homology-modeling–based mutagenesis studies (19, 20). In addition, in the recently published cryo-EM structures (10), most OCT ligands coresolved bound to OCTs either in the outward-open or inward-open states, were in close proximity to Phe244 (Fig. 1*A*). Further evidence for an important function of Phe244 comes from phylogenetic transporter comparisons which demonstrate that Phe244 is conserved among related transporters (Fig. 1*B*). The related OCT2 and OCT3 but also the mainly zwitterion transporting organic cation transporters novels, and the organic anion transporters 1 to 3 show all aromatic amino acids at this position. As for OCT1, also OCT3 and organic cation transporters novel 1 have phenylalanine at the corresponding codon whereas the other transporters have either a tryptophan or tyrosine.

**Figure 1:**
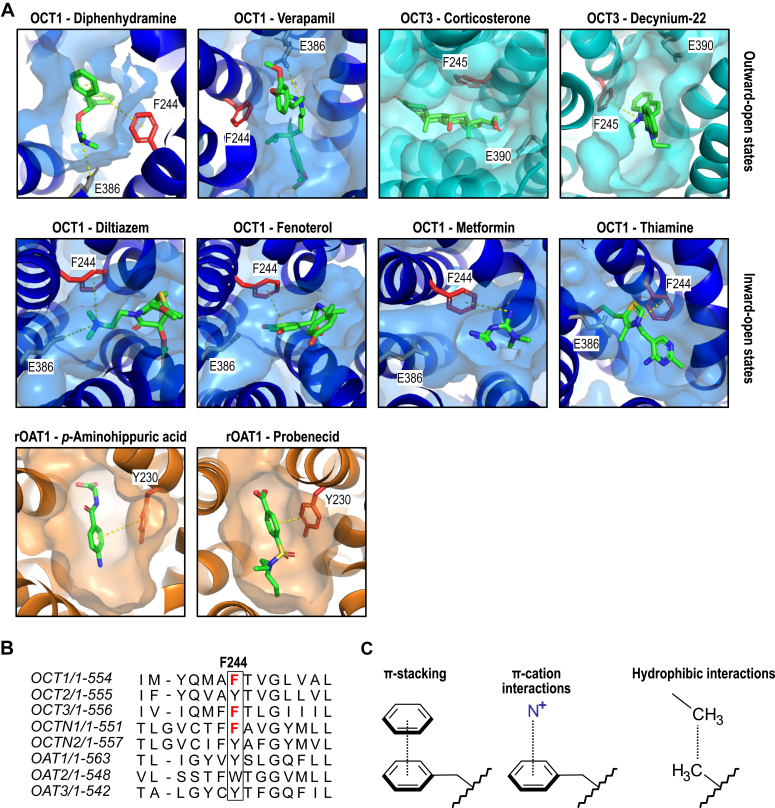
**Phenylalanine 244 as essential part of the ligand binding pocket of OCTs.***A*, ligand binding sites of multiple OCT1, OCT3, and OAT1 ligands coresolved within the transporter protein by cryo-EM (10, 11, 12, 26). Phenylalanine 244 (250 for OCT3) is shown in *red*. Glutamate 386 (390 for OCT3) is illustrated in *gray*. *Yellow dashed lines* indicate important interactions suggested by the respective reference publications. *B*, sequence alignment of SLC22 transporters for organic cations (OCTs), zwitterions (organic cation transporters novels), and anions (OATs) shows an aromatic amino acid at position 244. Alignment was done using the MUSCLE algorithm (40) and visualized with Jalview (41). *C*, phenylalanine may interact with small molecules *via* π-stacking or π–cation interactions, whereas alanine residues can only engage in hydrophobic interactions (21). SLC, solute carrier.

In addition to this structural evidence, functional studies demonstrated that removing the aromatic ring by substitution of phenylalanine by alanine might have strong substrate-specific effects. Phenylalanine may engage ligands mostly *via* relatively strong π-electron stacking forces or π–electron cation interactions (21). Removal of the phenolic ring *via* substitution to alanine disrupts both interactions and only allows hydrophobic contact (Fig. 1*C*). Consequences of the Phe244Ala substitution ranged from complete loss of transport activity for the antidiabetic drug metformin (10) up to even increased transport rates in a stereoselective manner of the beta-adrenergic agonist fenoterol (20). However, functional consequences of the Phe244Ala substitution were only investigated for a few OCT1 substrates.

Taken together, with these numerous hints on a potentially critical role of Phe244 on substrate binding, we comprehensively characterized the uptake of 144 previously known OCT1 substrates. These substrates were characterized by uptake ratios of at least 2-fold in OCT1-overexpressing cells over empty vector–transfected cells in earlier studies tested at a substrate concentration of 2.5 μM (8, 22, 23, 24, 25). In addition, we also investigated whether the inhibitory potencies of known OCT1 inhibitors may be affected by this substitution. Our data should improve the molecular understanding of ligand–OCT1 interactions.

## Results

**Figure 2:**
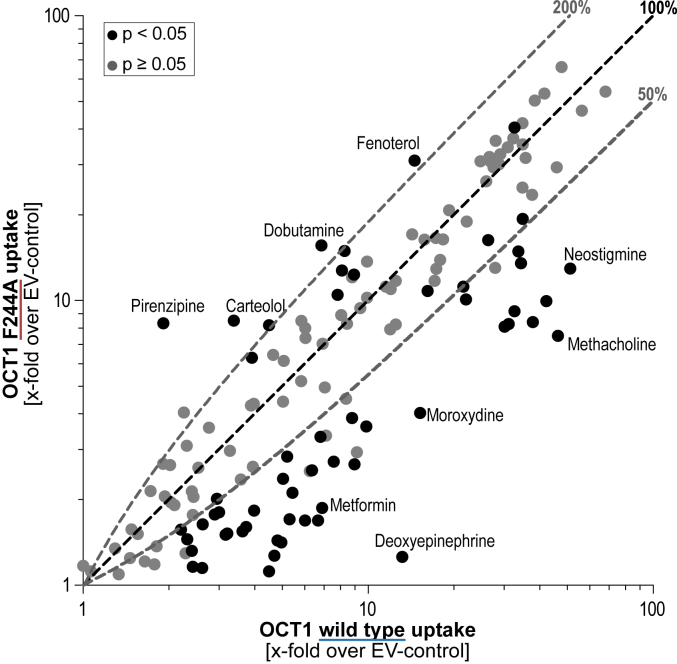
**Correlation of uptake data.** Correlation of uptake ratios of the WT and Phe244Ala variant of OCT1. Uptake ratio is shown as x-fold increase uptake into transporter-overexpressing cells over an empty vector (EV)-transfected control cell line. The *dashed lines* indicate uptake ratios of 200%, 100%, and 50% compared to the WT, respectively. Statistical significance in the difference of WT and Phe244Ala transport was determined by unpaired Student’s *t* test and is presented by *gray and dark dots* using a *p* value of 0.05 as threshold. OCT, organic cation transporter.

Analysis of the uptake of 144 previously known substrates *via* OCT1 Phe244Ala compared to the WT revealed a highly diverse pattern. The effects ranged from strongly reduced transport activities to as low as 10% of WT activity for methacholine, metformin, and mescaline, to up to 3.2- and even 8.0-fold increased uptake rates for carteolol and pirenzepine, respectively. Generally, 57 substrates (39%) showed reduced uptake below 50% while 15 substrates (10%) showed at least 50% increase in transport rates (Figs. 2 and S1). Out of those, six substrates (4%) showed uptake rates increased to 200% compared to the WT.

### Phe244Ala effects are related to substrate’s molecular weight, lipophilicity, and ring count

We analyzed a number of physicochemical properties as possible determinants of the effect of the Phe244Ala substitution. Molecular weight, lipophilicity, ring count, and molecular shape of substances were significantly different between substrates with altered transport activity for the Phe244Ala variant compared to the WT of OCT1 (Tables 1 and S4 for all calculated properties). Substrates, which showed at least 200% uptake compared to the WT, had on average a higher molecular weight, were more lipophilic as indicated by higher logD_7.4_ values, and had a higher ring count than those with decreased uptake rates below 50%. Additionally, substrates with reduced transport by the Phe244Ala variant compared to the WT were less complex as illustrated by higher molecular shape indices.

**Table 1:** Basic chemical properties of substrates with increased, normal, or reduced uptake by the Phe244Ala variant

Property	Uptake increased to >200%	No major change 200% ≥ uptake ≥ 50%	Uptake decreased to < 50%
N	6	81	57
Molecular weight∗∗∗	312.7 (244.3–383.4)	281.8 (137.2–484.7)	217.4 (109.1–441.6)
logD_7.4_∗∗	0.48 (−0.89 to 1.49)	−0.69 (−3.72 to 2.11)	−1.17 (−5.11 to 1.41)
Total polar surface area	74.8 (24.4–119,3)	60.4 (0–120.4)	64.8 (12.0–180.7)
Charge_7.4_	0.83 (0–1)	1 (0–2)	0.95 (0–2)
Ring count∗∗	2.3 (2–4)	2.7 (0–7)	1.5 (0–5)
H-bond donors	2.6 (1–5)	1.7 (0–4)	2.2 (0–4)
H-bond acceptors	5.2 (2–8)	4.2 (1–7)	4.0 (1–8)
Molecular shape index∗∗∗^a^	0.58 (0.46–0.73)	0.57 (0.42–0.78)	0.64 (0.4–0.81)

Data is presented as arithmetic means (minimum − maximum). Descriptors were tested for statistical significance in cross-group comparison across all three groups using the Kruskal−Wallis test (∗*p* ≤ 0.05, ∗∗*p* ≤ 0.01, and ∗∗∗*p* ≤ 0.001).

a Molecular shape index: Values of 1.0 represent molecules organized as perfect chains and the smaller the value the more rings and bridges are within the molecule. Molecular shape index was calculated by the DataWarrior suite (35).

### Phe244Ala alters stereoselective cell uptake by OCT1

**Figure 3:**
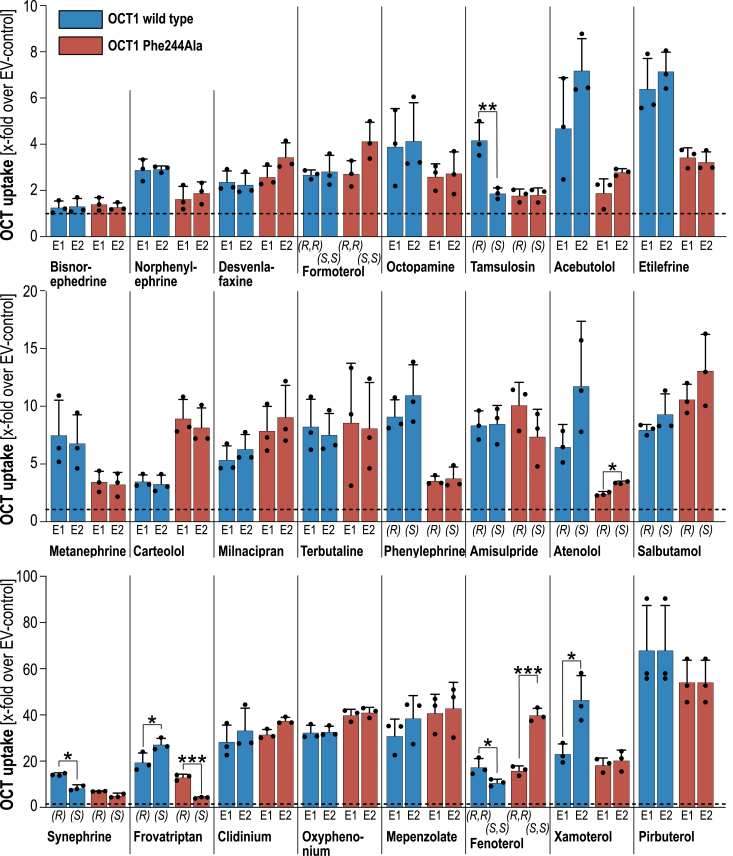
**Stereoselective cell uptake by OCT1 WT and Phe244Ala variant.** HEK293 cells overexpressing OCT1 WT, the Phe244Ala variant, and empty vector–transfected controls were incubated with 2.5 μM racemic substance for 2 min. Intracellular concentrations were quantified after chiral chromatography by mass spectrometry and are presented as mean ± SD of three independent experiments. *Dashed, horizontal lines* indicate uptake ratios of 1. Whenever the absolute steric conformation of the enantiomers was unknown, the enantiomers were termed according to their elution as enantiomer-1 (E1) and enantiomer-2 (E2). The *asterisks* indicate statistically significant differences between two enantiomers with unpaired Student’s *t* test and ∗*p* < 0.05, ∗∗*p* < 0.01, and ∗∗∗*p* < 0.001. OCT, organic cation transporter.

Among the studied substrates were many chiral substances. We analyzed effects of the Phe244Ala substitution on stereoselectivity in OCT1 transport for 24 racemic substrates (Fig. 3). OCT1 WT and the Phe244Ala variant showed statistically significant stereoselective uptake of only five and three substances, respectively. Interestingly, synephrine, tamsulosin, and xamoterol which showed stereoselective uptake *via* WT OCT1 lacked any stereoselective transport by the Phe244Ala variant. In contrast, fenoterol and frovatriptan showed opposite stereoselectivities between the WT and its variant. Nevertheless, most substances were not transported with any stereoselectivity.

### Transport kinetic effects of the Phe244Ala substitution

**Figure 4:**
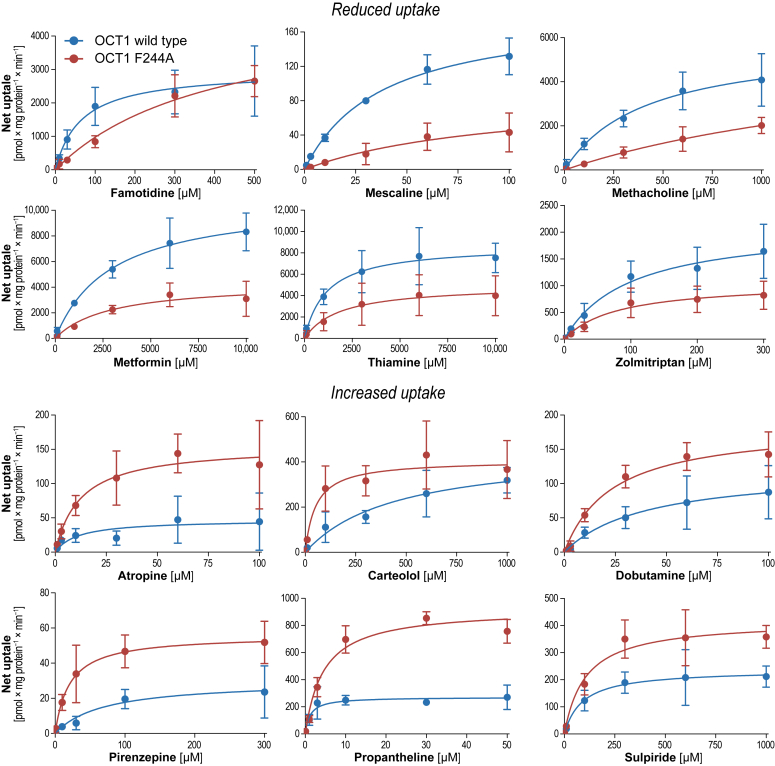
**Uptake kinetics by OCT1 WT and Phe244Ala substitution**. HEK293 cells overexpressing the WT OCT1, OCT1-Phe244Ala, and empty vector–transfected control cells were incubated with increasing drug concentrations for 2 min. Shown is the net uptake as mean ± SD of three independent experiments. OCT, organic cation transporter.

To investigate whether the pleiotropic effects of the Phe244Ala substitution are the results of altered affinity or rather transport capacity, we analyzed 12 substances by concentration-dependent transport kinetic experiments. We tested six which showed increased and another six with decreased uptake ratios in the previous screening (Fig. 4).

Out of those substrates, which showed reduced transport in the initial screening, metformin, thiamine, and zolmitriptan were characterized by a reduced transport capacity. Famotidine and methacholine showed more similar maximum transport capacities but transport by the Phe244Ala variant showed higher K_m_ values (Table 2). Mescaline transport was characterized by higher apparent affinity K_m_ and transport capacity v_max_ for the WT transporter than the Phe244Ala variant. For those with increased transport, all substrates except carteolol showed increased maximum transport capacities. Dobutamine and pirenzepine showed in addition also lower K_m_ values for the Phe244Ala variant. Carteolol was the only substrate with similar v_max_ but 8.4-fold lower K_m_ for the Phe244Ala variant than the WT transporter.

**Table 2:** Transport kinetic parameters

Substance	Variant	K_m_ [μM]	v_max_ [pmol × mg protein^−1^ × min^−1^]	Cl_int_ [ml × g Protein^−1^ × min^−1^]
Substrates with reduced transport in Phe244Ala compared to WT				
Famotidine	WT	64.7 ± 50.2	2963 ± 653	45.8 ± 45.7
	Phe244Ala	404 ± 433	4906 ± 2746	12.1 ± 19.8
Mescaline	WT	38.2 ± 14.3	185 ± 28.1	4.84 ± 2.55
	Phe244Ala	97.0 ± 168	88.4 ± 89.2	0.91 ± 2.50
Methacholine	WT	413 ± 314	5836 ± 1850	14.1 ± 15.2
	Phe244Ala	1999 ± 3184	6056 ± 6930	3.03 ± 8.29
Metformin	WT	2912 ± 1627	10,813 ± 2176	3.71 ± 2.82
	Phe244Ala	2828 ± 2728	4361 ± 1497	1.54 ± 2.02
Thiamine	WT	1161 ± 1029	8717 ± 1893	7.51 ± 8.28
	Phe244Ala	1844 ± 2864	4986 ± 2302	2.70 ± 5.44
Zolmitriptan	WT	98.5 ± 87.8	2126 ± 704	21.6 ± 26.4
	Phe244Ala	80.0 ± 78.0	1065 ± 357	13.3 ± 17.5
Substrates with increased transport in Phe244Ala compared to WT				
Atropine	WT	11.1 ± 22.7	47.0 ± 25.6	4.24 ± 11.0
	Phe244Ala	12.3 ± 12.2	155 ± 41.5	12.7 ± 15.9
Carteolol	WT	449 ± 438	453 ± 191	1.01 ± 1.41
	Phe244Ala	52.0 ± 45.4	407 ± 67.6	7.82 ± 8.13
Dobutamine	WT	40.1 ± 57.7	122 ± 72.6	3.03 ± 6.16
	Phe244Ala	23.5 ± 14.2	185 ± 37.8	7.88 ± 6.38
Pirenzepine	WT	69.3 ± 103	30.0 ± 14.5	0.43 ± 0.85
	Phe244Ala	20.4 ± 12.9	55.8 ± 8.52	2.74 ± 2.15
Propanthelin	WT	1.26 ± 1.12	271 ± 50.3	224 ± 251
	Phe244Ala	4.51 ± 2.16	923 ± 118	205 ± 124
Sulpiride	WT	89.2 ± 85.2	237 ± 49.7	2.65 ± 3.09
	Phe244Ala	104 ± 69	418 ± 65	4.01 ± 3.27

Data are given as arithmetic mean ± SD of three independent experiments.

### Phe244Ala reduces binding affinity of OCT1 inhibitors

**Figure 5:**
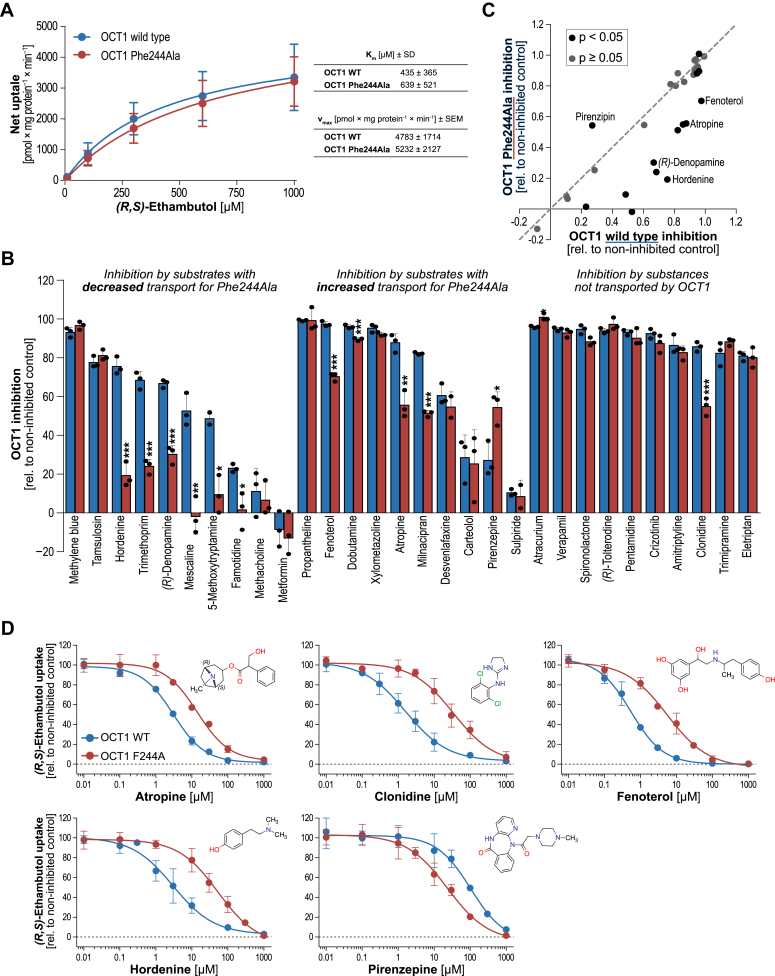
**Inhibition of OCT1 Phe244Ala variant.***A*, uptake kinetics of *(R,S)*-ethambutol by OCT1 WT and its Phe244Ala variant. HEK293 cells overexpressing the WT OCT1, OCT1 Phe244Ala, and empty vector–transfected control cells were incubated with increasing concentrations of *(R,S)*-ethambutol for 2 min. Shown is the net uptake as mean ± SD of three independent experiments. *B*, inhibition screening of selected OCT1 ligands. 20 μM inhibitors were coincubated with 2 μM *(R,S)*-ethambutol for 5 min. Empty vector–transfected cells were used as a control to account for passive diffusion. Data are presented as percentage inhibition values with mean ± SD of three independent experiments. *Asterisks* indicate the statistical significance of the differences between the inhibition of WT and mutant (Student’s *t* test; ∗*p* < 0.05, ∗∗*p* < 0.01, and ∗∗∗*p* < 0.001). *C*, correlation of percentage inhibition values. *D*, concentration-dependent inhibition by selected inhibitors. Mean values ± SD of three independent experiments are shown. OCT, organic cation transporter.

Among OCT1 substrates tested here, most were weak OCT1 inhibitors and high inhibition was mainly found for certain high-affinity substrates such as fenoterol and dobutamine. Interestingly, the Phe244Ala substitution led to increased uptake rates of many of these substrates which could be explained by a reduced binding affinity resulting in faster substrate release from inward-facing transporters. To test, whether binding affinities of these substances as well as of other OCT1 inhibitors is affected by the Phe244Ala substitution, we analyzed whether the inhibitory potencies might be affected. As model substrate for inhibition experiments we used the *(R,S)*-stereoisomer of ethambutol, since it showed similar uptake kinetics for the WT and Phe244Ala variant of OCT1 (Fig. 5*A*).

We tested 30 substances for transporter inhibition and selected ten substrates which showed reduced or increased uptake ratios, respectively, as well as ten known, nontransported OCT1 inhibitors. In an initial screening, multiple substances showed reduced transporter inhibition for the Phe244Ala variant of OCT1 compared to the WT (Fig. 5, *B* and *C*). Especially, clonidine, hordenine, mescaline, and trimethoprim showed strongly reduced inhibition of the mutant compared to the WT transporter—regardless of whether those substances showed increased or decreased uptake in our uptake screening or are not transported at all as with clonidine. Only pirenzepine showed more potent inhibition of the Phe244Ala variant. Inhibitory potencies of analyzed inhibitors differed by up to 21-fold as with clonidine (Fig. 5*D* and Table 3).

**Table 3:** IC_50_ values of transporter inhibition

Inhibitor	Transporter variant	IC_50_ [μM]	95% CI
Atropine	WT	3.08	2.57–3.67
	Phe244Ala	14.1	9.72–20.4
Clonidine	WT	1.62	1.04–2.52
	Phe244Ala	34.3	17.8–65.9
Fenoterol	WT	0.541	0.435–0.672
	Phe244Ala	5.53	3.82–8.01
Hordenine	WT	3.00	1.78–5.06
	Phe244Ala	53.2	29.0–97.7
Pirenzepine	WT	104	53.6–200
	Phe244Ala	23.3	14.2–38.3

## Discussion

Recently published cryo-EM structures of OCTs have fundamentally improved the molecular understanding of transporter–ligand interactions (10, 11, 12). OCT structures analyzed without substrates and in complex with several bound substrates and inhibitors in different states of the transport cycle have partially explained the promiscuous transport known already for a long time. Most cationic substrates interact with glutamic acid 386 for binding and subsequent translocation (10). In addition, several aromatic amino acids are part of the ligand binding site. Remarkably, in a recent publication of the structure of the rat organic anion transporter, the authors identified an “aromatic cage” around the ligand binding site which is highly conserved within the SLC22 family (26). In our study, we highlight the pleiotropic role of the aromatic amino acid phenylalanine 244 for OCT1 transport and inhibition.

Substitution of phenylalanine to alanine disrupts any pi-electron mediated aromatic interactions between the amino acid at codon 244 and the ligands. For OCT1, we could show that the Phe244Ala substitution had strong substrate-specific effects. Importantly, it was shown previously that the Phe244Ala substitution does not affect the membrane abundance of OCT1 (16). In our single dose uptake screening, most substances showed reduced uptake rates. However, numerous substrates were taken up more efficiently by the mutant compared to the WT (Fig. 2). Both groups were separated by significant differences in their physicochemical properties. Substrates which showed increased transport rates had on average a higher molecular weight, higher lipophilicity reflected by logD_pH7.4_ values, and more aromatic rings than those which showed reduced transport (Table 1). In addition to the basic physicochemical properties of the substrates, we also analyzed possible effects of the Phe244Ala substitution on stereoselectivity of OCT1. For several substrates, such as fenoterol, frovatriptan, and xamoterol, only one enantiomer of the racemic mixtures showed altered transport by the Phe244Ala variant. This indicates specific molecular interactions in a stereoselective manner for some substances although most substrates showed no altered stereoselectivity. However, the number of stereoselectively analyzed chiral substrates was too low to allow drawing general conclusions how the Phe244Ala mediates stereoselective effects.

**Figure 6:**
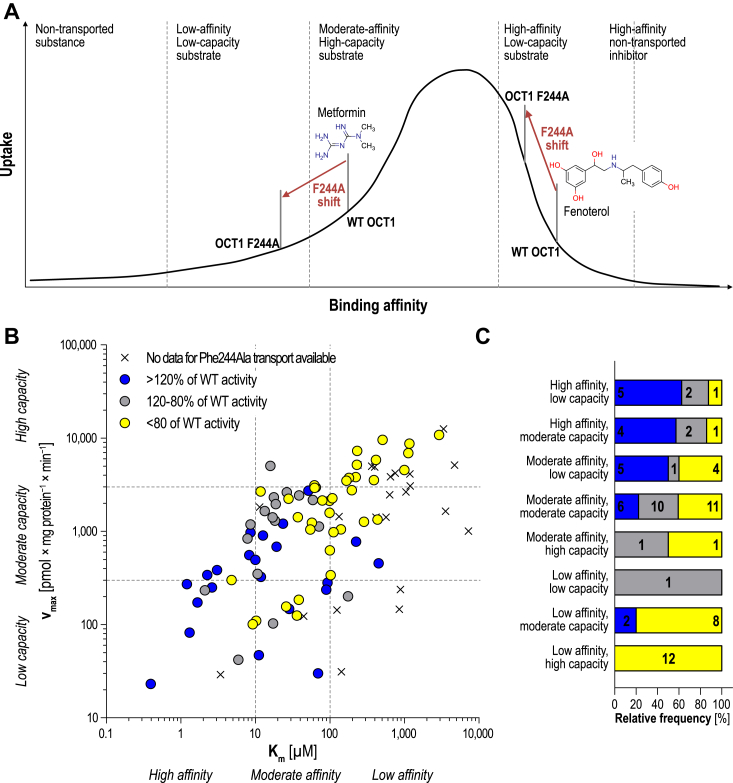
**Uptake-affinity model explains substrate-specific effects of OCT1 Phe244Ala substitution.***A*, model of uptake and binding affinity. Increasing binding affinity results in increased uptake rates until a maximum is reached after which further increasement of affinity transforms a substrate into a nontransported inhibitor. The Phe244Ala substitution might reduce affinity which results in decreased uptake for low affinity and increased uptake for formerly high affinity substrates. *B*, correlation of transport capacity v_max_ and apparent affinity K_m_ and frequency distribution of altered transport activity. Transport kinetic data was taken from this study, published literature (4, 8, 9, 14, 20, 24, 27, 28, 36, 37, 38, 39) and previously unpublished data (Fig. S2). Colors indicate increased or reduced uptake ratios within our screening. *C*, relative distribution of increased, normal, and decreased activity of the Phe244Ala variant. *Numbers within the stacked bars* indicate the absolute numbers. OCT, organic cation transporter.

Generally, the pleiotropic effects might be explained by a simple affinity-uptake model although other factors might contribute to the observed phenotype of the Phe244Ala substitution (Fig. 6).

It is reasonable to assume that without any affinity to the transporter, a substance shows no uptake. With increasing affinity, the uptake rates also increase. In addition, also the inhibitory potency of a substrate increases with rising affinity. Consequently, substances with infinitively high affinity will act as nontransported inhibitors. As a result, increasing the affinity of a substance will lead to a certain optimum in transport. Further increase of affinity beyond this point will strongly decrease the carrier-mediated uptake as very tight binding contradicts substrate release. Except for pirenzepine, we can assume that reduced binding affinity might explain the pleiotropic effects of the Phe244Ala substitution. This is supported by the inhibition data since most substances, regardless of whether they show increased or reduced uptake, showed lower inhibitory potencies for the Phe244Ala variant than the WT (Table 3). According to this simple model, high-affinity substrates such as atropine, dobutamine, and fenoterol show increased transport rates due to a moderate reduction in affinity, whereas the reduced binding affinity for low-affinity substrates results in even less transport as, for example, with hordenine and metformin (Fig. 6*A*).

Supportive evidence for this hypothesis comes also from a comparison to published transport-kinetic data for OCT1 (Fig. 6, *B* + C). Although K_m_ values may not entirely reflect actual binding affinities, they can be used as semiquantitative approximation of transporter binding. High-affinity substrates showed more frequently increased transport, whereas with decreasing affinity, more substrates showed reduced uptake rates. Especially substrates classified as low-affinity, high-capacity showed exclusively reduced uptake rates within our screening.

Pirenzepine is the only studied substrates which shows increased uptake as well as increased transporter inhibition for the Phe244Ala variant of OCT1. A hypothesis explaining this finding could be that pirenzepine belongs with its nonflexible pyridobenzodiazepine moiety to the larger OCT1 substrates. In addition to the disruption of the critical intermolecular interactions illustrated in Figure 1*C*, the phenylalanine to alanine substitution might also lead to an enlargement of the ligand binding cavity since alanine lacks the bulky phenolic ring. Accordingly, this might allow pirenzepine to occupy different binding poses within the cavity leading to increased affinity and subsequent also increased uptake. However, experimental structural data would be required to evaluate this hypothesis. This illustrates impressively how the cryo-EM–driven structural insights and traditional ligand-based biochemical activity studies complement each other. On the one side, the hypothesis for this study was driven by the recently published insights by OCT cryo-EM structures. On the other side, the functional data generated here further triggers novel research questions to be addressed through cryo-EM.

Altogether, our study demonstrates the important role of phenylalanine 244 for the substrate translocation *via* OCT1. It also highlights that testing multiple, structurally diverse substrates is necessary to fully elucidate the function of amino acids within the binding pocket of polyspecific OCT1. The use of single “model substrate” only cannot provide a complete picture of the structure and function of highly polyspecific membrane transporters such as OCT1.

## Experimental procedures

### Test substances

All investigated substances were purchased from Angene Chemicals, Cayman Chemical, Roche Pharma, Santa Cruz Biotechnology, Sigma-Aldrich, and Toronto Research Chemicals with purities of at least 95% according to the respective manufacturers. A list of all substances including the respective manufacturer and its catalog number is provided in Table S1. Chiral compounds were tested as racemate if not otherwise specified either by the drug name or by assigning a chiral configuration within the drug SMILES.

### Cell lines and cell culture conditions

All experiments were carried out using transporter-overexpressing HEK293 cells. Cell lines were generated previously by stable transfection using the Flp-In system (Thermo Fisher Scientific). Detailed generation and validation of these cell lines has been reported before (20, 27). Cells were cultivated in Dulbecco’s Modified Eagle’s medium supplemented with 10% fetal calf serum, 100 U/ml penicillin, and 100 μg/ml streptomycin. Cells were passaged twice a week and kept in culture for no longer than 30 passages.

### Immunofluorescence staining of OCT1 Phe244Ala variant

To confirm correct membrane localization, OCT1 WT and Phe244Ala mutant–expressing cells were stained by immunofluorescence. For this, 100.000 cells were plated in a poly-d-lysine coated 4-well Nunc Lab-Tec II Chamber slides (Thermo Fisher Scientific) 48 h in advance. For immunofluorescence staining, cells were washed twice with Dulbecco’s PBS (D-PBS; Thermo Fisher Scientific), and fixation was done with 4% paraformaldehyde in D-PBS for 20 min at room temperature. Subsequently, cells were washed three times with D-PBS before permeabilization was done with D-PBS/0.1% Triton X-100 (Carl Roth) for 15 min at room temperature. After three subsequent washing steps with D-PBS/0.1% Tween-20 (Sigma-Aldrich, referred to as D-PBST from here on), blocking was done with 0.1% Tween-20, 1% bovine serum albumin (BSA; Sigma-Aldrich), and 20% normal goat serum (NGS; Abcam) diluted in D-PBS for 60 min. After this, incubation with primary antibodies was done overnight at 4 °C using monoclonal mouse anti-OCT1 (Clone ID: 2C5; Novus Biologicals, Cat# NBP1-51684) and monoclonal rabbit anti-sodium-potassium ATPase (Clone ID: EP1845Y; Abcam, Cat# AB76020) both in the dilution of 1:100 in D-PBS supplemented with 0.1% Tween-20, 1% BSA, and 5% NGS. On the next day, cells were washed three times with D-PBST and then stained with goat Alexa Fluor 555 polyclonal anti-mouse IgG and goat Alexa Fluor 633 polyclonal anti-rabbit IgG (Thermo Fisher Scientific), both diluted 1:500 in D-PBS with 0.1% Tween-20, 1% BSA and 1.5% NGS and incubated on cells, for 60 min at room temperature. Afterward, cells were washed three times with D-PBST and counterstaining was done using 1 μg/ml 4′,6-diamidin-2-phenylindol (DAPI; Sigma-Aldrich) dissolved in PBS for 3 min at room temperature. Finally, cells were washed once with PBS and slides were mounted using Roti-Mount FluorCare (Carl Roth). Images were taken with a Leica DMi8 microscope (Leica Microsystems) equipped with a STEDYcon module (Abberior instruments). Images acquired in confocal mode using a 63 × oil immersion objective with identical acquisition settings for all images.

### *In vitro* transport experiments

For uptake studies, 300,000 HEK293 cells were plated in each well of 24-well plates precoated with poly-d-lysine 48 prior to the experiment. On the day of the experiment, cells were washed once with prewarmed to 37 °C HBSS+ (Hank’s balanced salt solution supplemented with 10 mM Hepes, both Sigma-Aldrich). Uptake was then initiated by adding 2.5 μM substrate dissolved in HBSS+ and incubated for 2 min at 37 °C. Uptake was stopped by adding ice-cold HBSS+ and cells were washed twice with ice-cold buffer before cell lysis was done using 80% acetonitrile containing an appropriate internal standard for eventual mass spectrometry. Relative quantification was done by comparison of transporter-overexpressing cells to empty vector–transfected controls. For uptake kinetic experiments, cells were incubated with increasing concentrations of substrate dissolved in HBSS+. Absolute quantification was done later by comparison to a standard curve in lysis buffer with known concentration.

### *In vitro* inhibition experiments

For cellular inhibition experiments, 300,000 HEK293 cells were plated in poly-d-lysine precoated 24 well plates 48 h in advance. To study transporter inhibition, cells were incubated with 2 μM *(R,S)*-ethambutol with and without 20 μM inhibitor both dissolved in HBSS+. Uptake of the model substrate was terminated after 5 min by aspiration of the buffer and cells were washed twice with ice-cold HBSS+ before cell lysis was done with 80% acetonitrile containing 10 ng/ml choline-d9 as internal standard for ethambutol concentration analysis.

### Liquid chromatography coupled to tandem mass spectrometry

Intracellular drug concentrations were detected and quantified by HPLC coupled to mass spectrometry. The Shimadzu Nexera HPLC system was composed of a CBM-20A controller, a LC-30AD pump, a CTO-20AC column oven and an SIL-30AC autosampler (all Shimadzu). Achiral compound separation was done on a Brownlee SPP RP-Amide column with 4.6 × 100 mm inner dimensions and a particle size of 2.7 μm as well as a preceding Phenomenex C-18 guard column. Chromatography was carried out in reversed-phase mode using aqueous 0.1 (v/v) formic acid with an acetonitrile:methanol ratio 6:1 (v:v) in total concentrations ranging from 3% to 50% (v/v) of solvent/water. Substance-specific conditions were used to adjust the retention times of the analytes. For separation, the column oven temperature was set to 40 °C, and the flow rate was adjusted to 300 or 400 μl/min. Substance detection was done with an API 4000 tandem mass spectrometer (AB SCIEX), and peak integration and quantification was performed using the Analyst software (https://sciex.com/products/software/analyst-software; AB SCIEX, version 1.6.2).

For chiral chromatography, substances were separated either on a CHIRALPAK AGP HPLC column (100 × 2.1 mm inner dimensions, 5 μm particle size; Sigma-Aldrich), a CHIRALPAK CBH HPLC column (100 × 3 mm inner dimensions, 5 μm particle size; Sigma-Aldrich), or an Astec chirobiotic T column (150 × 2.1 mm, 5 μm particle size; Sigma-Aldrich) with the corresponding guard columns. Chromatography was carried out with in reversed-phase mode with an aqueous mobile phase buffered with ammonium acetate and supplemented with 2-propanol or methanol as organic modifiers (Table S2). Order of elution of enantiomers was obtained either by reference literature (28, 29, 30, 31, 32, 33) or by injecting single enantiomers. Whenever there was no information available, the first eluting enantiomer was referred to as E1 and the second as E2. Bambuterol, milnacipran, and proguanil were used as appropriate internal standards for analysis on the AGP, CBH, and chirobiotic T columns, respectively. Mass spectrometry detection parameters are summarized in Table S3.

## Calculations

Transport ratios were determined as relative fold-increase of the uptake in transporter-overexpressing cells over empty vector–transfected control cells. For transport kinetic experiments, absolute substrate quantification was done by signal comparison to a standard curve with known concentrations. Net uptake was then determined by subtracting the uptake into empty vector–transfected cells from transporter-overexpressing cells. Net uptake was then plotted against the substrate concentration [S], and the data was analyzed by nonlinear regression analysis following the Michaelis–Menten equation *v* = *v*_max_ × [*S*]/(*K*_m_ + [*S*]) using GraphPad Prism (https://www.graphpad.com/; Version 5.01 for Windows, GraphPad Software). V_max_ defines the maximum transport velocity, K_m_ refers to the substrate concentration required to reach half v_max_. The intrinsic clearance Cl_int_ is the ratio of v_max_ over K_m_.

In inhibition experiments, transporter activity was calculated as it follows:

$$\%\;\text{OCT}1\;\text{activity}=\frac{\left[{\text{Substrate}}_{\text{inhibited}}\right]-\left[{\text{Substrate}}_{\text{EV}}\right]}{\left[{\text{Substrate}}_{\text{non}-\text{inhibited}}\right]-\left[{\text{Substrate}}_{\text{EV}}\right]}$$

Substrate_inhibited_ is the uptake of ethambutol into transporter-overexpressing cells in presence of an inhibitor, whereas substrate_non-inhibited_ refers to the ethambutol uptake without coincubation with an inhibitor. Substrate_EV_ refers to the ethambutol uptake into empty vector–transfected control cells. Percent inhibition values where then calculated as in the following:

%transporterinhibition=100%−transporteractivity

In concentration-dependent inhibition experiments, the transporter activity was plotted against the log_10_ of inhibitor concentrations. The data was then analyzed by the following formula to determine IC_50_ values:

$$Y=Y_{\min}+\frac{\left(Y_{\max}-Y_{\min}\right)}{1+10^{\log_{10}\left(\text{IC}50-x\right)∙\text{hill}\;\text{slope}}}$$

*Y* describes the transporter activity, whereas *Y*_max_ is to the maximum and *Y*_min_ to the minimal transporter activity. X refers to the log_10_ of inhibitor concentrations, IC_50_ characterizes the half-maximal inhibitory concentration, and hill slope describes the slope factor. The regression was done using GraphPad Prism (Version 5.01 for Windows, GraphPad Software)

### Physicochemical property analysis

Two-dimensional structures of investigated drugs were stored as isomeric SMILES obtained from PubChem database (34). Based on this, logD values at pH 7.4 were calculated using MarvinSketch provided by Chemaxon (https://www.chemaxon.com). All other parameters were calculated using DataWarrior (35). All calculated chemical descriptors are listed in Table S4. This table also contains the mean uptake ratios for the WT and Phe244Ala OCT1.

## Data availability

All data generated or analyzed during this study are included in this published article.

## Supporting information

This article includes supporting information (4, 8, 9, 14, 20, 24, 27, 28, 36, 37, 38, 39).

## Conflicts of interest

The authors declare that they have no conflicts of interest with the contents of this article.
