# Supplementary material for: Comprehensive characterization of the OCT1 phenylalanine-244-alanine substitution reveals highly substrate-dependent effects on transporter function
Source: J Biol Chem. 2024 Sep 27;300(11):107835. doi: 10.1016/j.jbc.2024.107835 (PMC11602988; doi:10.1016/j.jbc.2024.107835)
Supplement: Supporting information [file mmc1.docx]

## Supporting information

Comprehensive characterization of the OCT1 phenylalanine-244-alanine substitution reveals highly substrate-dependent effects on transporter function

Carla Isabel Wittern^1^, Sophie Schröder^2^, Ole Jensen^1^, Jürgen Brockmöller^1^, Lukas Gebauer^1^

^1^ Institute of Clinical Pharmacology, University Medical Center Göttingen, D-37075 Göttingen, Germany

^2^ Department for Epigenetics and Systems Medicine in Neurodegenerative Diseases, German Center for Neurodegenerative Diseases (DZNE), D-37075 Göttingen, Germany

E-Mail: lukas.gebauer@med.uni-goettingen.de

**Table of contents**

Figure S1: Uptake ratios of wild-type OCT1 and its Phe244Ala variant

Figure S2: Unpublished wild-type transport kinetic uptake data for OCT1

Figure S3: Subcellular localization of wild-type OCT1 and Phe244Ala variant

Table S1: Test substances including drug SMILES, manufacturer, and catalogue number

Table S2: HPLC conditions for chiral separation of investigated substances

Table S3: Mass spectrometric detection parameters

Table S4: Physicochemical properties of investigated substances (separate file)


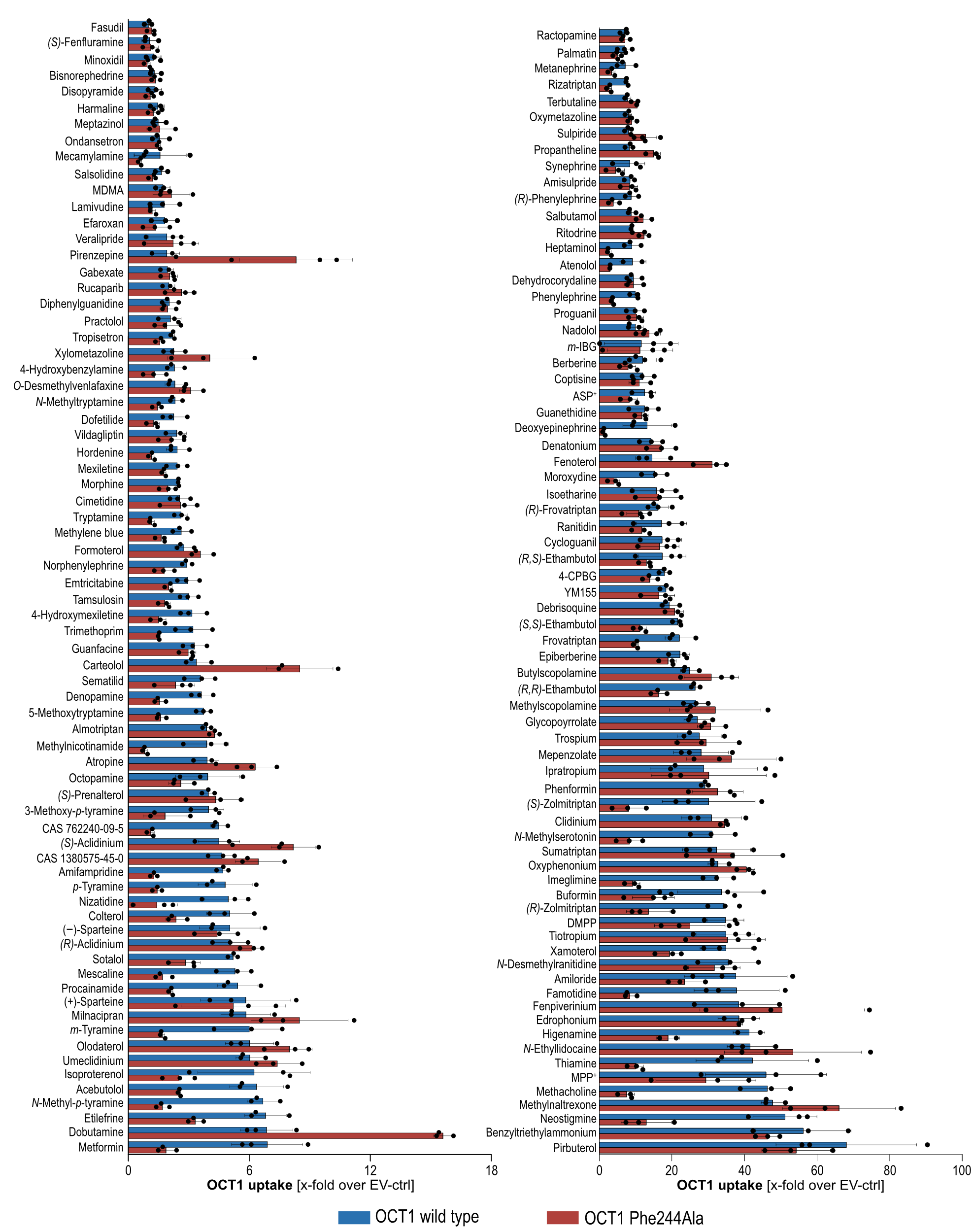


**Figure S1: Uptake ratios of wild-type OCT1 and its Phe244Ala variant**

HEK293 cells overexpressing OCT1 wild type, the PHE244ALA variant and empty-vector transfected controls were incubated with 2.5 µM substance for 2 min. Data is presented as mean ± SD of three independent experiments. Abbreviations: ASP+, 4-(4-(dimethylamino)styryl)-N-methylpyridinium; 4-CPBG, 1-(4-Chlorophenyl)biguanide; DMPP, Dimethylphenylpiperizinium; MDMA, 3,4-Methyl​enedioxy​methamphetamine; MPP^+^, 1-Methyl-4-phenylpyridinium.


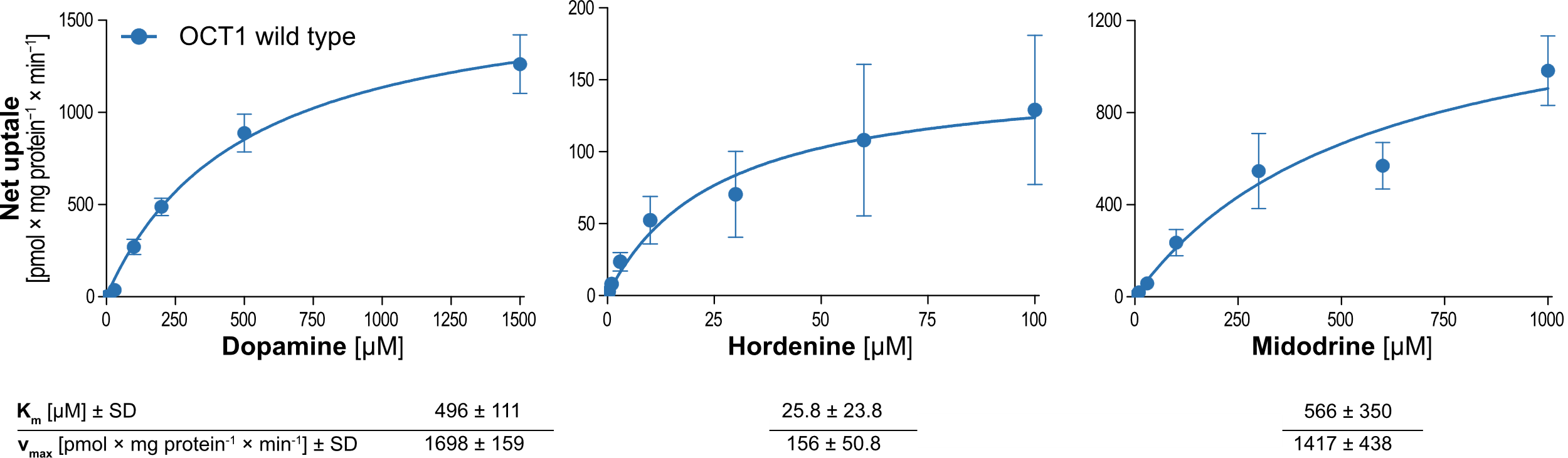


**Figure S2: Unpublished wild type transport kinetic uptake data for OCT1**

HEK293 cells overexpressing wild-type OCT1, and empty-vector transfected control cells were incubated with increasing concentrations of dopamine, hordenine, and midodrine for 2 min. Shown is the net uptake as mean ± SD of three independent experiments. Transport kinetic constants are reported below each plot.


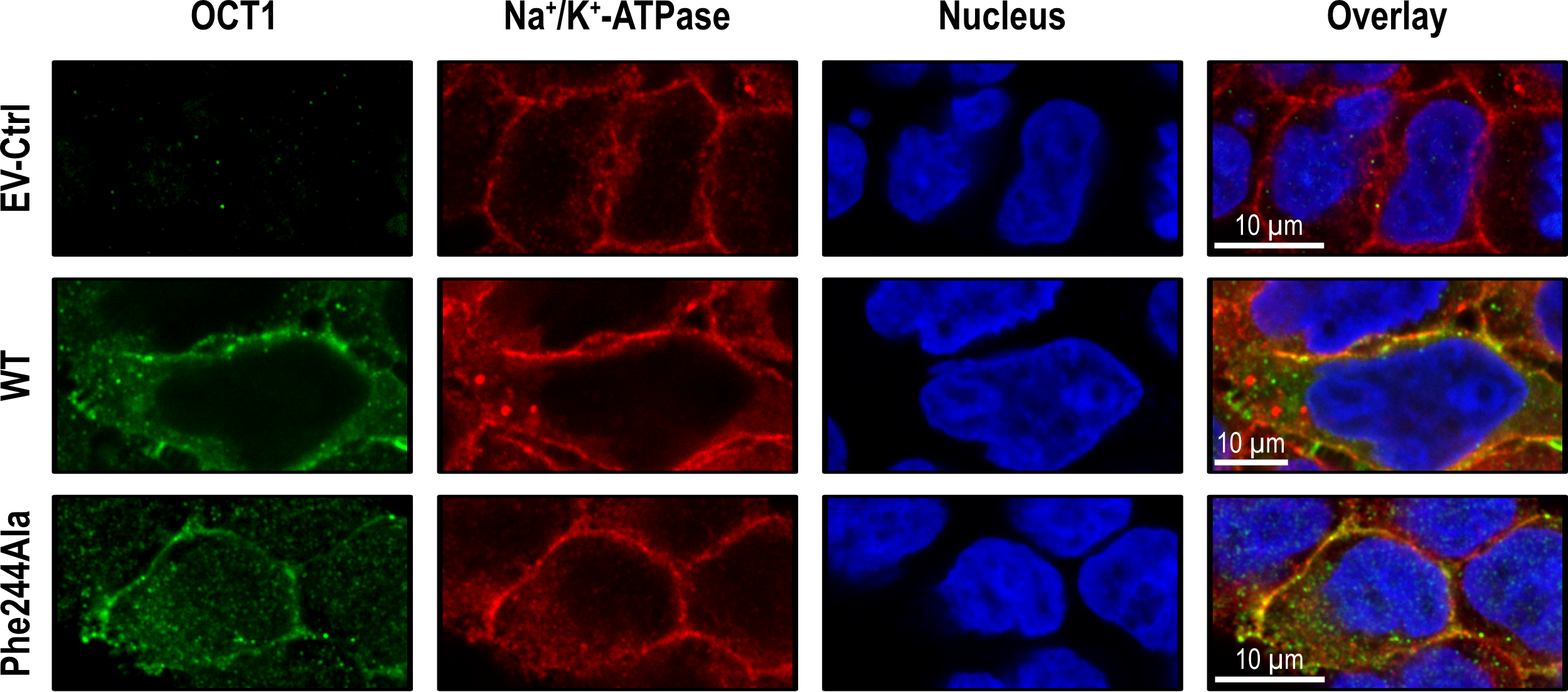


**Figure S3: Subcellular localization of wild-type OCT1 and Phe244Ala variant**

Stable-transfected cells with empty vector (EV) or OCT1 wild type (WT) and its Phe244Ala variant were stained with anti-OCT1 and compared to the staining of the Na^+^/K^+^-ATPase as marker of the plasma membrane. Colocalization of the OCT1 and Na^+^/K^+^-ATPase signals indicates correct membrane localization of the transporter.

**Table S1:** **Test substances including drug SMILES, manufacturer, and catalogue number**

| **Substance** | **Isomeric SMILES** | **Manufacturer** | **Cat#** |
| --- | --- | --- | --- |
| Acebutolol | CCCC(=O)NC1=CC(=C(C=C1)OCC(CNC(C)C)O)C(=O)C | Sigma^a^ | A3669-1G |
| Acetyl-beta-methylcholine | CC(C[N+](C)(C)C)OC(=O)C | Sigma^a^ | A2251-25G |
| *(R)*-Aclidinium | C1C[N+]2(CCC1[C@H](C2)OC(=O)C(C3=CC=CS3)(C4=CC=CS4)O)CCCOC5=CC=CC=C5 | Sigma^a^ | SML2868 |
| *(S)*-Aclidinium | C1C[N+]2(CCC1[C@@H](C2)OC(=O)C(C3=CC=CS3)(C4=CC=CS4)O)CCCOC5=CC=CC=C5 | TRC^b^ | A190190 |
| Almotriptan | CN(C)CCC1=CNC2=C1C=C(C=C2)CS(=O)(=O)N3CCCC3 | Sigma^a^ | SML1210-10MG |
| Amifampridine | C1=CN=CC(=C1N)N | Sigma^a^ | D-7148-1G |
| Amiloride | C1(=C(N=C(C(=N1)Cl)N)N)C(=O)N=C(N)N | Sigma^a^ | A7410-1G |
| Amisulpride | CCN1CCCC1CNC(=O)C2=CC(=C(C=C2OC)N)S(=O)(=O)CC | Sigma^a^ | A2729-10MG |
| ASP^+^ | C[N+]1=CC=C(C=C1)/C=C/C2=CC=C(C=C2)N(C)C | Sigma^a^ | 336408-1G |
| Atenolol | CC(C)NCC(COC1=CC=C(C=C1)CC(=O)N)O | Sigma^a^ | A7655-1G |
| Atropine | CN1[C@@H]2CC[C@H]1CC(C2)OC(=O)C(CO)C3=CC=CC=C3 | Sigma^a^ | A0132-1G |
| Benzyltriethylammonium | CC[N+](CC)(CC)CC1=CC=CC=C1 | Sigma^a^ | 146552-25G |
| Berberine | COC1=C(C2=C[N+]3=C(C=C2C=C1)C4=CC5=C(C=C4CC3)OCO5)OC | Sigma^a^ | B3251-5G |
| Bisnorephedrine | C1=CC=C(C=C1)C(CN)O | Sigma^a^ | A72405-10G |
| Buformin | CCCCN=C(N)N=C(N)N | Sigma^a^ | 2810052-25G |
| Butylscopolamine | CCCC[N+]1([C@@H]2CC(C[C@H]1[C@H]3[C@@H]2O3)OC(=O)[C@H](CO)C4=CC=CC=C4)C | Sigma^a^ | S7882-1G |
| Carteolol | CC(C)(C)NCC(COC1=CC=CC2=C1CCC(=O)N2)O | Sigma^a^ | BP567 |
| CAS 1380575-45-0 | CC1=CC(=C(C=C1C2CCNCC2)OC(C)C)N | Angene^c^ | AG0017WW |
| CAS 762240-09-5 | C1CNCCC1N2C=CC=N2 | Angene^c^ | AG008O9A |
| Cimetidine | CC1=C(N=CN1)CSCCNC(=NC)NC#N | Sigma^a^ | C4522-5G |
| 1-(4-Chlorophenyl)-biguanide | C1=CC(=CC=C1N=C(N)N=C(N)N)Cl | Sigma^a^ | S447366-1G |
| Clidinium | C[N+]12CCC(CC1)C(C2)OC(=O)C(C3=CC=CC=C3)(C4=CC=CC=C4)O | Sigma^a^ | C0414-1G |
| Colterol | CC(C)(C)NCC(C1=CC(=C(C=C1)O)O)O | TRC^b^ | C215850 |
| Coptisine | C1C[N+]2=C(C=C3C=CC4=C(C3=C2)OCO4)C5=CC6=C(C=C51)OCO6 | TRC^b^ | C685500 |
| Cycloguanil | CC1(N=C(N=C(N1C2=CC=C(C=C2)Cl)N)N)C | SCBT^d^ | sc-207470 |
| Debrisoquine | C1CN(CC2=CC=CC=C21)C(=N)N | Sigma^a^ | D1306-100MG |
| Dehydrocorydaline | CC1=C2C=CC(=C(C2=C[N+]3=C1C4=CC(=C(C=C4CC3)OC)OC)OC)OC | Sigma^a^ | SML3501-5MG |
| Denatonium | CC[N+](CC)(CC1=CC=CC=C1)CC(=O)NC2=C(C=CC=C2C)C | Sigma^a^ | D5765-1G |
| *(R)*-Denopamine | COC1=C(C=C(C=C1)CCNC[C@@H](C2=CC=C(C=C2)O)O)OC | Sigma^a^ | D7815-5MG |
| Deoxyepinephrine | CNCCC1=CC(=C(C=C1)O)O | TRC^b^ | D232920 |
| *N*-Desmethylranitidine | CNCC1=CC=C(O1)CSCCNC(=C[N+](=O)[O-])NC | TRC^b^ | D292160-1MG |
| *O*-Desmethylvenlafaxine | CN(C)CC(C1=CC=C(C=C1)O)C2(CCCCC2)O | Sigma^a^ | 93867-50MG |
| Dimethylphenyl-piperazinium | C[N+]1(CCN(CC1)C2=CC=CC=C2)C | Sigma^a^ | D5891-1G |
| 1,3-Diphenylguanidine | C1=CC=C(C=C1)NC(=NC2=CC=CC=C2)N | Sigma^a^ | D207756-500G |
| Disopyramide | CC(C)N(CCC(C1=CC=CC=C1)(C2=CC=CC=N2)C(=O)N)C(C)C | Sigma^a^ | D6035-1G |
| Dobutamine | CC(CCC1=CC=C(C=C1)O)NCCC2=CC(=C(C=C2)O)O | Sigma^a^ | D0676-10MG |
| Dofetilide | CN(CCC1=CC=C(C=C1)NS(=O)(=O)C)CCOC2=CC=C(C=C2)NS(=O)(=O)C | CAY^e^ | CAY15045-10 mg |
| Edrophonium | CC[N+](C)(C)C1=CC(=CC=C1)O | SCBT^d^ | sc-207610 |
| Efaroxan | CCC1(CC2=CC=CC=C2O1)C3=NCCN3 | Sigma^a^ | E3263-10MG |
| Emtricitabine | C1[C@H](O[C@H](S1)CO)N2C=C(C(=NC2=O)N)F | Sigma^a^ | PHR2120-500MG |
| Epiberberine | COC1=C(C=C2C(=C1)CC[N+]3=C2C=C4C=CC5=C(C4=C3)OCO5)OC | TRC^b^ | E578300-10MG |
| *(R,R)*-Ethambutol | CC[C@H](CO)NCCN[C@H](CC)CO | TRC^b^ | E889805 |
| *(R,S)*-Ethambutol | CC[C@H](CO)NCCN[C@@H](CC)CO | TRC^b^ | E67805 |
| *(S,S*)-Ethambutol | CC[C@@H](CO)NCCN[C@@H](CC)CO | Sigma^a^ | E4630 |
| *N*-Ethyllidocaine | CC[N+](CC)(CC)CC(=O)NC1=C(C=CC=C1C)C | Sigma^a^ | L5783-50MG |
| Etilefrine | CCNCC(C1=CC(=CC=C1)O)O | SCBT^d^ | sc-294579a |
| Famotidine | C1=C(N=C(S1)N=C(N)N)CSCC/C(=N/S(=O)(=O)N)/N | SCBT^d^ | sc-205691-500MG |
| Fasudil | C1CNCCN(C1)S(=O)(=O)C2=CC=CC3=C2C=CN=C3 | Sigma^a^ | CDS021620-10MG |
| *(S)*-Fenfluramine | CCN[C@@H](C)CC1=CC(=CC=C1)C(F)(F)F | TRC^b^ | F247596 |
| Fenoterol | CC(CC1=CC=C(C=C1)O)NCC(C2=CC(=CC(=C2)O)O)O | Sigma^a^ | F1016 |
| Fenpiverinium | C[N+]1(CCCCC1)CCC(C2=CC=CC=C2)(C3=CC=CC=C3)C(=O)N | SCBT^d^ | sc-211504-10MG |
| Formoterol | CC(CC1=CC=C(C=C1)OC)NCC(C2=CC(=C(C=C2)O)NC=O)O | Sigma^a^ | PHR2703 |
| *(R)*-Frovatriptan | CN[C@@H]1CCC2=C(C1)C3=C(N2)C=CC(=C3)C(=O)N | Sigma^a^ | SML1291-10MG |
| Frovatriptan | CNC1CCC2=C(C1)C3=C(N2)C=CC(=C3)C(=O)N | Sigma^a^ | 1286404-15MG |
| Gabexate | CCOC(=O)C1=CC=C(C=C1)OC(=O)CCCCCN=C(N)N | Sigma^a^ | SML2964-5MG |
| Glycopyrrolate | C[N+]1(CCC(C1)OC(=O)C(C2CCCC2)(C3=CC=CC=C3)O)C | Sigma^a^ | SML0025-5MG |
| Guanethidine | C1CCCN(CCC1)CCN=C(N)N | Sigma^a^ | BP181-100MG |
| Guanfacine | C1=CC(=C(C(=C1)Cl)CC(=O)N=C(N)N)Cl | Sigma^a^ | G1043-10MG |
| Harmaline | CC1=NCCC2=C1NC3=C2C=CC(=C3)OC | Sigma^a^ | 51330-1G |
| Heptaminol | CC(CCCC(C)(C)O)N | Sigma^a^ | Y000063 |
| Higenamine | C1CNC(C2=CC(=C(C=C21)O)O)CC3=CC=C(C=C3)O | Sigma^a^ | SML2313-5MG |
| Hordenine | CN(C)CCC1=CC=C(C=C1)O | Sigma^a^ | 04476-100MG |
| 4-Hydroxy benzylamine | C1=CC(=CC=C1CN)O | Sigma^a^ | CDS003156-1G |
| 4-Hydroxy mexiletine | CC1=CC(=CC(=C1OCC(C)N)C)O | TRC^b^ | H948020 |
| Imeglimin | C[C@@H]1N=C(NC(=N1)N(C)C)N | Sigma^a^ | SML2401-5MG |
| *m*-Iodobenzylguanidine | C1=CC=C(C=C1)CN(C(=N)N)I | Sigma^a^ | I9890-5MG |
| Ipratropium | CC(C)[N+]1([C@@H]2CC[C@H]1CC(C2)OC(=O)C(CO)C3=CC=CC=C3)C | SCBT^d^ | sc-252911-250MG |
| Isoetharine | CCC(C(C1=CC(=C(C=C1)O)O)O)NC(C)C | Sigma^a^ | I3639-1G |
| Isoproterenol | CC(C)NCC(C1=CC(=C(C=C1)O)O)O | Sigma^a^ | I5627-5G |
| Lamivudine | C1[C@H](O[C@H](S1)CO)N2C=CC(=NC2=O)N | Sigma^a^ | PHR1365-1G |
| Mecamylamine | CC1(C2CCC(C2)C1(C)NC)C | Sigma^a^ | M9020-5MG |
| Mepenzolate | C[N+]1(CCCC(C1)OC(=O)C(C2=CC=CC=C2)(C3=CC=CC=C3)O)C | Sigma^a^ | M5651-5G |
| Meptazinol | CCC1(CCCCN(C1)C)C2=CC(=CC=C2)O | Sigma^a^ | M2824-10MG |
| Mescaline | COC1=CC(=CC(=C1OC)OC)CCN | Sigma^a^ | M-257 |
| Metanephrine | CNCC(C1=CC(=C(C=C1)O)OC)O | TRC^b^ | M258760 |
| Metformin | CN(C)C(=N)N=C(N)N | Sigma^a^ | D5035-25G |
| Methylene blue | N(C)C1=CC2=C(C=C1)N=C3C=CC(=[N+](C)C)C=C3S2 | Sigma^a^ | M9140-25G |
| Methylnaltrexone | C[N+]1(CC[C@]23[C@@H]4C(=O)CC[C@]2([C@H]1CC5=C3C(=C(C=C5)O)O4)O)CC6CC6 | Sigma^a^ | SML0277-5MG |
| 1-Methylnicotinamide | C[N+]1=CC=CC(=C1)C(=O)N | Sigma^a^ | SML0704-10MG |
| Methylscopolamine | C[N+]1([C@@H]2CC(C[C@H]1[C@H]3[C@@H]2O3)OC(=O)[C@H](CO)C4=CC=CC=C4)C | Sigma^a^ | S8502-1G |
| *N*-Methylserotonin | CNCCC1=CNC2=C1C=C(C=C2)O | TRC^b^ | M326595 |
| *N*-Methyltryptamine | CNCCC1=CNC2=CC=CC=C21 | TRC^b^ | M331920 |
| *N*-Methyl-*p*-tyramine | CNCCC1=CC=C(C=C1)O | SCBT^d^ | sc-391686-1G |
| 3,4-Methylendioxy-*N*-methylamphetamin | CC(CC1=CC2=C(C=C1)OCO2)NC | Sigma^a^ | M-013 |
| 3-Methoxy-p-tyramine | COC1=C(C=CC(=C1)CCN)O | TRC^b^ | M332320-100MG |
| 5-Methoxy tryptamine | COC1=CC2=C(C=C1)NC=C2CCN | Sigma^a^ | 286583-100MG |
| Mexiletine | CC1=C(C(=CC=C1)C)OCC(C)N | Sigma^a^ | M2727-25G |
| Milnacipran | CCN(CC)C(=O)C1(CC1CN)C2=CC=CC=C2 | SCBT^d^ | sc-204086 |
| Minoxidil | C1CCN(CC1)C2=NC(=N)N(C(=C2)N)O | Sigma^a^ | M4145-25MG |
| Moroxydine | C1COCCN1C(=N)N=C(N)N | Sigma^a^ | 278610-1G |
| Morphine | CN1CC[C@]23[C@@H]4[C@H]1CC5=C2C(=C(C=C5)O)O[C@H]3[C@H](C=C4)O | Sigma^a^ | M8777 |
| MPP^+^ | C[N+]1=CC=C(C=C1)C2=CC=CC=C2 | Sigma^a^ | D048-1G |
| Nadolol | CC(C)(C)NCC(COC1=CC=CC2=C1C[C@@H]([C@@H](C2)O)O)O | Sigma^a^ | N1892-1G |
| Neostigmine | CN(C)C(=O)OC1=CC=CC(=C1)[N+](C)(C)C | Sigma^a^ | N2001-1G |
| Nizatidine | CN/C(=C\[N+](=O)[O-])/NCCSCC1=CSC(=N1)CN(C)C | Sigma^a^ | N7035-5G |
| Norphenylephrine | C1=CC(=CC(=C1)O)C(CN)O | Sigma^a^ | 113727-10G |
| Octopamine | C1=CC(=CC=C1C(CN)O)O | Sigma^a^ | O0250-1G |
| Olodaterol | CC(C)(CC1=CC=C(C=C1)OC)NC[C@@H](C2=C3C(=CC(=C2)O)NC(=O)CO3)O | Sigma^a^ | O262000 |
| Ondansetron | CC1=NC=CN1CC2CCC3=C(C2=O)C4=CC=CC=C4N3C | Sigma^a^ | 03639-10MG |
| Oxymetazoline | CC1=CC(=C(C(=C1CC2=NCCN2)C)O)C(C)(C)C | Sigma^a^ | O2378-5G |
| Oxyphenonium | CC1=CC(=C(C(=C1CC2=NCCN2)C)O)C(C)(C)C | Sigma^a^ | O5501-1G |
| Palmatin | COC1=C(C2=C[N+]3=C(C=C2C=C1)C4=CC(=C(C=C4CC3)OC)OC)OC | Sigma^a^ | SMB00472-100MG |
| Phenformin | C1=CC=C(C=C1)CCN=C(N)N=C(N)N | Sigma^a^ | P7045-10G |
| *(R)*-Phenylephrine | CNC[C@@H](C1=CC(=CC=C1)O)O | Sigma^a^ | P6126-5G |
| Phenylephrine | CNCC(C1=CC(=CC=C1)O)O | TRC^b^ | P320635 |
| Pirbuterol | CC(C)(C)NCC(C1=NC(=C(C=C1)O)CO)O | Sigma^a^ | 32142-10MG |
| Pirenzepine | CN1CCN(CC1)CC(=O)N2C3=CC=CC=C3C(=O)NC4=C2N=CC=C4 | Sigma^a^ | Y0000038 |
| Practolol | CC(C)NCC(COC1=CC=C(C=C1)NC(=O)C)O | Sigma^a^ | SML1462-5MG |
| *(S)*-Prenalterol | CC(C)NC[C@@H](COC1=CC=C(C=C1)O)O | SCBT^d^ | sc-280023A-5MG |
| Procainamide | CCN(CC)CCNC(=O)C1=CC=C(C=C1)N | Sigma^a^ | P9391-25G |
| Proguanil | CC(C)N=C(N)/N=C(\N)/NC1=CC=C(C=C1)Cl | Sigma^a^ | G7048 |
| Propantheline | CC(C)[N+](C)(CCOC(=O)C1C2=CC=CC=C2OC3=CC=CC=C13)C(C)C | Sigma^a^ | P8891-5G |
| Ractopamine | CC(CCC1=CC=C(C=C1)O)NCC(C2=CC=C(C=C2)O)O | Sigma^a^ | 34198-100MG |
| Ranitidine | CN/C(=C\[N+](=O)[O-])/NCCSCC1=CC=C(O1)CN(C)C | Sigma^a^ | R101-1G |
| Ritodrine | C[C@@H]([C@@H](C1=CC=C(C=C1)O)O)NCCC2=CC=C(C=C2)O | Sigma^a^ | R0758-250MG |
| Rizatriptan | CN(C)CCC1=CNC2=C1C=C(C=C2)CN3C=NC=N3 | Sigma^a^ | SML0247 |
| Rucaparib | CNCC1=CC=C(C=C1)C2=C3CCNC(=O)C4=C3C(=CC(=C4)F)N2 | Sigma^a^ | PZ0036-5MG |
| Salbutamol | CC(C)(C)NCC(C1=CC(=C(C=C1)O)CO)O | Sigma^a^ | S8260-25MG |
| Salsolidine | CC1C2=CC(=C(C=C2CCN1)OC)OC | Sigma^a^ | S100005 |
| Sematilide | CCN(CC)CCNC(=O)C1=CC=C(C=C1)NS(=O)(=O)C | Sigma^a^ | S0323-10MG |
| Sotalol | CC(C)NCC(C1=CC=C(C=C1)NS(=O)(=O)C)O | SCBT^d^ | sc-203699-10MG |
| *(−)*-Sparteine | C1CCN2C[C@H]3C[C@@H]([C@H]2C1)CN4[C@H]3CCCC4 | Sigma^a^ | S2251-10G |
| *(+)*-Sparteine | C1CCN2C[C@@H]3C[C@H]([C@H]2C1)CN4[C@H]3CCCC4 | Sigma^a^ | 92052-1G |
| Sulpiride | CCN1CCCC1CNC(=O)C2=C(C=CC(=C2)S(=O)(=O)N)OC | Sigma^a^ | S8010-25G |
| Sumatriptan | CNS(=O)(=O)CC1=CC2=C(C=C1)NC=C2CCN(C)C | Sigma^a^ | S1198-50MG |
| Synephrine | CNCC(C1=CC=C(C=C1)O)O | Sigma^a^ | S0752-5G |
| Tamsulosin | CCOC1=CC=CC=C1OCCNC(C)CC2=CC(=C(C=C2)OC)S(=O)(=O)N | Sigma^a^ | Y0000653 |
| Terbutaline | CC(C)(C)NCC(C1=CC(=CC(=C1)O)O)O | Sigma^a^ | T2528-1G |
| Thiamine | CC1=C(SC=[N+]1CC2=CN=C(N=C2N)C)CCO | Sigma^a^ | T1270-25G |
| Tiotropium | C[N+]1([C@@H]2CC(C[C@H]1[C@H]3[C@@H]2O3)OC(=O)C(C4=CC=CS4)(C5=CC=CS5)O)C | Sigma^a^ | SML2008-10MG |
| Trimethoprim | COC1=CC(=CC(=C1OC)OC)CC2=CN=C(N=C2N)N | Sigma^a^ | PHR1056-1G |
| Tropisetron | CN1[C@@H]2CC[C@H]1CC(C2)OC(=O)C3=CNC4=CC=CC=C43 | SCBT^d^ | sc-204930-10MG |
| Trospium | C1CC[N+]2(C1)[C@@H]3CC[C@H]2CC(C3)OC(=O)C(C4=CC=CC=C4)(C5=CC=CC=C5)O | Sigma^a^ | PHR3378-200MG |
| Tryptamine | C1=CC=C2C(=C1)C(=CN2)CCN | Sigma^a^ | 246557-5G |
| *m*-Tyramine | C1=CC(=CC(=C1)O)CCN | TRC^b^ | T898505-100MG |
| *p*-Tyramine | C1=CC(=CC=C1CCN)O | Sigma^a^ | T90344-5G |
| Umeclidinium | C1C[N+]2(CCC1(CC2)C(C3=CC=CC=C3)(C4=CC=CC=C4)O)CCOCC5=CC=CC=C5 | TRC^b^ | U710000 |
| Veralipride | COC1=CC(=CC(=C1OC)C(=O)NCC2CCCN2CC=C)S(=O)(=O)N | Sigma^a^ | T7632-5MG |
| Vildagliptin | C1C[C@H](N(C1)C(=O)CNC23CC4CC(C2)CC(C4)(C3)O)C#N | Sigma^a^ | SML2302-50MG |
| Xamoterol | C1COCCN1C(=O)NCCNCC(COC2=CC=C(C=C2)O)O | TRC^b^ | X499808 |
| Xylometazoline | CC1=CC(=CC(=C1CC2=NCCN2)C)C(C)(C)C | Sigma^a^ | X6000-5G |
| YM155 | CC1=[N+](C2=C(N1CCOC)C(=O)C3=CC=CC=C3C2=O)CC4=NC=CN=C4 | BYT^f^ | BYT-ORB1306455 |
| *(R)*-Zolmitriptan | CN(C)CCC1=CNC2=C1C=C(C=C2)C[C@@H]3COC(=O)N3 | Sigma^a^ | Y0001986 |
| *(S)-*Zolmitriptan | CN(C)CCC1=CNC2=C1C=C(C=C2)C[C@H]3COC(=O)N3 | Sigma^a^ | SML0248 |

^a^ Sigma-Aldrich Chemie GmbH, Darmstadt, Germany

^b^ Toronto Research Chemicals, Toronto, Canada

^c^ Angene Chemicals, London, United Kingdom

^d^ Santa-Cruz Biotechnology, Dallas, USA

^e^ Cayman Chemical, Ann Arbor, USA

^f^ Biorbyt, Cambridge, United Kingdom

**Table S2** HPLC conditions for chiral separation of investigated substances

| **Substance** | **HPLC column** | **Mobile phase composition** | **Flow rate**  [μL min^-^1] | **Temperature**  [°C] | **Retention time**  [min; order of elution] |
| --- | --- | --- | --- | --- | --- |
| Acebutolol | Chiralpak CBH | 10 mM NH_4_Ac, pH 5.8; 10% IPA | 300 | 22 | 4.16 / 12.50 [R-S] (40) |
| Amisulpride | Chiralpak CBH | 10 mM NH_4_Ac, pH 5.8; 10% IPA | 300 | 25 | 5.35 / 5.74 [S-R] |
| Atenolol | Chiralpak CBH | 10 mM NH_4_Ac, pH 5.8; 10% IPA | 300 | 22 | 3.75 / 5.41 [R-S] (39) |
| Carteolol | Chiralpak CBH | 10 mM NH_4_Ac, pH 5.0; 10% IPA | 500 | 25 | 3.03 / 5.05 [1-2] |
| Clidinium | Chiralpak AGP | 10 mM NH_4_Ac, pH 6.8; 10% IPA | 200 | 25 | 11.43 / 12.15 [1-2] |
| Desvenlafaxine | Chiralpak CBH | 10 mM NH_4_Ac, pH 5.8; 10% IPA | 300 | 22 | 3.84 / 4.16 [1-2] |
| Etilefrine | ChirobioticT | 20 mM NH_4_Ac, pH 4.5; 92% MeOH | 400 | 25 | 7.49 / 7.87 [1-2] |
| Fenoterol | Chiralpak CBH | 10 mM NH_4_Ac, pH 5.8; 10% IPA | 500 | 22 | 4.36 / 5.23 [RR-SS] (37) |
| Formoterol | Chiralpak CBH | 10 mM NH_4_Ac, pH 5.8; 10% IPA | 300 | 22 | 6.14 / 6.66 [RR-SS] |
| Frovatriptan | Chiralpak CBH | 10 mM NH_4_Ac, pH 5.0; 10% IPA | 400 | 25 | 5.87 / 7.82 [S-R] |
| Homatropine | Chiralpak AGP | 10 mM NH_4_Ac, pH 6.8; 10% IPA | 200 | 25 | 7.22 / 7.66 [1-2] |
| Metanephrine | Chiralpak CBH | 10 mM NH_4_Ac, pH 5.8; 10% IPA | 300 | 22 | 4.05 / 4.48 [1-2] |
| Metaproterenol | ChirobioticT | 20 mM NH_4_Ac, pH 4.5; 92% MeOH | 400 | 25 | 6.14 / 7.07 [1-2] |
| Mepenzolate | Chiralpak AGP | 10 mM NH_4_Ac, pH 6.8; 10% IPA | 200 | 25 | 10.08 / 10.65 [1-2] |
| Milnacipran | Chiralpak AGP | 10 mM NH_4_Ac, pH 6.8; 10% IPA | 200 | 25 | 8.64 / 8.97 [1-2] |
| Oxyphenonium | Chiralpak AGP | 10 mM NH_4_Ac, pH 5.8; 10% IPA | 200 | 25 | 7.99 / 9.41 [1-2] |
| Phenylephrine | Chiralpak CBH | 10 mM NH_4_Ac, pH 5.8; 10% IPA | 400 | 22 | 2.91 / 3.24 [R-S] (35) |
| Pirbuterol | Chiralpak CBH | 10 mM NH_4_Ac, pH 5.8; 10% IPA | 300 | 25 | 3.37 / 3.83 [1-2] |
| Salbutamol | ChirobioticT | 20 mM NH_4_Ac, pH 4.5; 95% MeOH | 400 | 25 | 8.75 / 9.58 [R-S] (38) |
| Synephrine | Chiralpak CBH | 10 mM NH_4_Ac, pH 5.8; 10% IPA | 400 | 22 | 2.86 / 3.35 [R-S] (36) |
| Tamsulosin | Chiralpak CBH | 10 mM NH_4_Ac, pH 5.0; 10% IPA | 500 | 25 | 3.41 / 6.74 [R-S] |
| Terbutaline | ChirobioticT | 20 mM NH_4_Ac, pH 4.5; 92% MeOH | 400 | 25 | 5.89 / 7.13 [1-2] |
| Xamoterol | Chiralpak CBH | 10 mM NH_4_Ac, pH 5.8; 10% IPA | 300 | 22 | 4.72 / 5.84 [1-2] |

AGP, α1-glycoprotein; CBH, Cellobiohydrolase; IPA, isopropyl alcohol; MeOH, methanol; NH_4_Ac, ammonium acetate

**Table S3:** Mass spectrometry detection parameters

| **Compound** | **RT**  **[min]** | **Mass Q1**  **[Da]** | **Mass Q3**  **[Da]** | **DP**  **[V]** | **CE**  **[V]** | **CXP**  **[V]** | **Internal standard** |
| --- | --- | --- | --- | --- | --- | --- | --- |
| **3% organic additive** (96.9% H2O, 0.1 % formic acid, 2.6% acetonitrile, 0.4% methanol) | | | | | | | |
| Amifampridine | 2.9 | 110.0 | 92.9  (82.9) | 76 | 30 (27) | 17 (15) | Buformin |
| Bisnorephridine | 4.3 | 137.89 | 119.91  (76.86) | 29 | 12  (40) | 8  (14) | Buformin |
| Buformin | 4.0 | 157.91 | 60.9  (47.0) | 36 | 35 (66) | 10  (8) | Choline-d9 |
| CAS 1380575-45-0 | 5.6 | 249.2 | 84.0  (166.1) | 75 | 23  (19) | 16  (10) | Buformin |
| CAS 762240-09-5 | 3.9 | 151.9 | 84.0  (55.0) | 56 | 21  (39) | 16  (10) | Buformin |
| Colterol | 6.0 | 226.148 | 152.0  (208.1) | 70 | 21  (15) | 12  (14) | Buformin |
| Deoxyepinephrine | 4.05 | 168.109 | 137.1  (91.1) | 56 | 17  (35) | 8  (16) | Buformin |
| Edrophonium | 4.7 | 167.2 | 137.0  (139.0) | 76 | 37  (23) | 8  (9) | Buformin |
| Ethambutol | 2.5 | 205.2 | 116.1  (55.1) | 66 | 21  (45) | 6  (10) | Buformin |
| Etilefrine | 7.68 | 182.124 | 164  (91) | 51 | 17  (37) | 10  (6) | Buformin |
| Guanethidine | 2.9 | 199.144 | 140.1  (86.0) | 60 | 25  (23) | 8  (16) | Buformin |
| Heptaminol | 5.25 | 146.22 | 128.1  (96) | 56 | 13  (23) | 8  (12) | Buformin |
| Hordenine | 4.88 | 166.14 | 121  (77) | 51 | 21  (47) | 8  (10) | Buformin |
| Imeglimin | 4.5 | 156.178 | 113.1  (71.1) | 71 | 23  (39) | 8  (14) | Buformin |
| Isoproterenol | 3.3 | 212.2 | 194  (151) | 41 | 15  (23) | 12  (10) | Buformin |
| Lamivudine | 4.4 | 230.0 | 111.9  (95.0) | 46 | 17 (53) | 6  (18) | Buformin |
| L-Phenylephrine | 4.06 | 168.21 | 91  (77) | 41 | 30  (56) | 11  (4) | Buformin |
| Metformin | 2.7 | 130.0 | 71.0 | 40 | 35 | 10 | Buformin |
| Methacholine | 3.5 | 160.107 | 101.0  (99.9) | 50 | 17  (15) | 18  (6) | Buformin |
| Methanephrine | 3.5 | 198.1 | 180  (165) | 41 | 13  (25) | 12  (10) | Buformin |
| m-Tyramine | 4.55 | 138.1 | 121  (77) | 31 | 15  (37) | 8  (14) | Buformin |
| 3-Methoxy-p-tyramine | 4.92 | 168.1 | 150.9  (119) | 41 | 13  (25) | 10  (8) | Buformin |
| Methylnicotinamide | 2.7 | 137.0 | 94.1  (78.0) | 65 | 29  (35) | 5  (14) | Buformin |
| Moroxydine | 3.1 | 172.151 | 60.2  (69.0) | 81 | 27  (41) | 10  (12) | Buformin |
| Norphenylephrine | 3.3 | 154.18 | 136  (91.1) | 39 | 11  (29) | 8  (16) | Buformin |
| Nizatidine | 6.6 | 332.2 | 155.0  (131.0) | 71 | 27  (35) | 10  (10) | Buformin |
| N-Methyl-p-Tyramine | 4.49 | 152.1 | 121.2  (103.1) | 51 | 17  (31) | 8  (6) | Buformin |
| Octopamine | 3.0 | 154.1 | 136  (91) | 36 | 11  (29) | 8  (16) | Buformin |
| Phenylephrine | 3.8 | 168.21 | 91  (77) | 41 | 30  (56) | 11  (4) | Buformin |
| Procainamide | 5.7 | 236.1 | 163.3  (120.1) | 55 | 22  (40) | 10 (7) | Buformin |
| p-Tyramine | 4.12 | 138.1 | 121.1 | 43 | 14 | 12 | Buformin |
| Serotonin | 3.03 | 177.1 | 160.1  (132) | 53 | 17  (30) | 10  (10) | Buformin |
| Sparteine | 5.6 | 138.1 | 98.2  (233.1) | 100 | 49  (36) | 8  (16) | Buformin |
| Synephrine | 3.1 | 167.691 | 149.9  (135) | 36 | 35  (35) | 12  (8) | Buformin |
| Thiamine | 2.8 | 177.1 | 122.1  (143.8) | 50 | 25  (19) | 10  (10) | Buformin |
| **8% organic additive** (91.9% H2O, 0.1 % formic acid, 6.9% acetonitrile, 1.1% methanol) | | | | | | | |
| Benzyl-triethylammonium | 5.7 | 193.32 | 92.1  (101.0) | 56 | 28  (23) | 17  (18) | Ranitidine-d6 |
| Cimetidine | 4.1 | 253.129 | 159.0  (95.2) | 61 | 21  (34) | 10  (18) | Ranitidine-d6 |
| Debrisoquine | 9.1 | 176.2 | 159.1  (134.2) | 80 | 25  (26) | 15  (15) | Ranitidine-d6 |
| Dimethylphenyl-piperazinium | 5.3 | 192.29 | 72.06  (58.0) | 96 | 34  (54) | 13  (10) | Ranitidine-d6 |
| Emtricitabine | 4.9 | 247.95 | 129.97 | 44 | 15 | 8 | Ranitidine-d6 |
| Famotidine | 4.4 | 338.5 | 189.0  (155.0) | 54 | 27  (43) | 12  (10) | Ranitidine-d6 |
| Frovatriptan | 5.18 | 244.3 | 170.1  (213) | 56 | 34  (19) | 10  (14) | Ranitidine-d6 |
| Hydroxybenzylamine | 3.1 | 124.11 | 107.0  (77.0) | 29 | 9  (35) | 6  (14) | Ranitidine-d6 |
| Isoetharine | 4.2 | 240.124 | 222.1  (123.1) | 61 | 19  (39) | 14  (22) | Ranitidine-d6 |
| Methylnaltrexone | 5.6 | 356.093 | 55.2  (284.1) | 98 | 61  (33) | 10  (18) | Ranitidine-d6 |
| Morphine | 3.2 | 286.2 | 201.1  (165.1) | 110 | 36  (54) | 15  (15) | Ranitidine-d6 |
| N-Methylserotonin | 4.07 | 191.101 | 160  (148) | 60 | 17  (17) | 10  (10) | Ranitidine-d6 |
| Pirbuterol | 3.7 | 241.3 | 167.2  (149.1) | 65 | 24  (30) | 15  (15) | Ranitidine-d6 |
| Ranitidine | 4.4 | 315.3 | 176.0  (130.1) | 65 | 24  (34) | 11  (8) | Ranitidine-d6 |
| Ranitidine-d6 | 4.4 | 321.2 | 176.0  (130.1) | 65 | 25  (35) | 15  (15) | - |
| (R)-Frovatriptan | 5.18 | 244.3 | 170.1  (213) | 56 | 34  (19) | 10  (14) | Ranitidine-d6 |
| Salbutamol | 3,8 | 240.2 | 148.2  (222.2) | 60 | 24  (24) | 15  (15) | Ranitidine-d6 |
| Salsolidine | 5.6 | 208.151 | 191.2  (160.1) | 66 | 21  (29) | 12  (12) | Ranitidine-d6 |
| Sematilide | 5.4 | 314.42 | 240.9  (162.1) | 79 | 27  (39) | 16  (10) | Ranitidine-d6 |
| Sotalol | 4.13 | 273.37 | 255.1  (133.1) | 61 | 17  (37) | 16  (8) | Ranitidine-d6 |
| (S)-Prenalterol | 4.3 | 226.28 | 149.1  (56.0) | 81 | 23  (39) | 10  (10) | Ranitidine-d6 |
| Terbutaline | 3.8 | 226.2 | 152.1  (107) | 60 | 23  (40) | 10  (10) | Ranitidine-d6 |
| Trimethoprim | 9.9 | 291.0 | 230.19  (123.01) | 94 | 33  (33) | 15  (8) | Ranitidine-d6 |
| Tryptamine | 5.77 | 161.06 | 144  (117) | 41 | 13  (33) | 10  (8) | Ranitidine-d6 |
| Vildagliptin | 4.65 | 304.286 | 154.1  (97.1) | 90 | 23  (43) | 10  (8) | Ranitidine-d6 |
| **20% organic additive** (79.9% H2O, 0.1 % formic acid, 17.2% acetonitrile, 2.8% methanol) | | | | | | | |
| Acebutolol | 4.3 | 337.243 | 116.0 | 91 | 31 | 8 | Fenoterol-d6 |
| Amiloride | 3.55 | 230.1 | 171  (60.2) | 66 | 23  (34) | 10  (10) | Fenoterol-d6 |
| Amisulpride | 3.8 | 370.2 | 242.1  (195.8) | 90 | 38  (52) | 15  (15) | Fenoterol-d6 |
| Atenolol | 2.9 | 267.2 | 145.2 | 130 | 38 | 10 | Fenoterol-d6 |
| Atropine | 2.9 | 290.2 | 142.2  (124.2) | 100 | 45  (33) | 12  (12) | Fenoterol-d6 |
| Butylscopolamine | 7.9 | 360.2 | 121.2 | 100 | 40 | 15 | Fenoterol-d6 |
| 1-(4-Chlorphenyl)biguanide | 4.9 | 212.09 | 153.02  (111.06) | 75 | 35  (52) | 10  (10) | Fenoterol-d6 |
| Carteolol | 3.5 | 293.219 | 237.3  (202.1) | 63 | 21  (30) | 16  (12) | Fenoterol-d6 |
| Cycloguanil | 3.3 | 252.2 | 195.1  (153.0) | 75 | 25  (41) | 10  (10) | Fenoterol-d6 |
| Denopamine | 3.98 | 318.106 | 165.1  (300.2) | 75 | 27  (17) | 10  (20) | Fenoterol-d6 |
| Desvenlafaxine | 3.5 | 264.3 | 107.2  (58.1) | 60 | 50  (47) | 6  (10) | Fenoterol-d6 |
| 1,3-Diphenylguanidine | 4.4 | 212.1 | 119.1  (77.0) | 96 | 29  (55) | 22  (4) | Fenoterol-d6 |
| Dobutamine | 3.14 | 302.202 | 137  (106.9) | 66 | 30  (37) | 10  (6) | Fenoterol-d6 |
| Dysopyramide | 2.7 | 340.242 | 239.1  (195.1) | 60 | 25  (41) | 16  (12) | Fenoterol-d6 |
| Efaroxan | 3.35 | 217.161 | 71.1  (91.2) | 80 | 32  (47) | 12  (16) | Fenoterol-d6 |
| Fenpiverinium | 6.1 | 338.5 | 239.1  (77.01) | 69 | 24  (111) | 16  (14) | Fenoterol-d6 |
| Fenoterol-d6 | 3.5 | 310.3 | 109.1  (141.0) | 70 | 40  (26) | 12  (12) | - |
| Formeterol | 7.8 | 345.2 | 149.1  (121.1) | 70 | 28  (42) | 15  (15) | Fenoterol-d6 |
| Guanfacine | 5.6 | 246.2 | 59.9  (229.15) | 36 | 32  (9) | 10  (6) | Fenoterol-d6 |
| Higenamine | 3.2 | 272.058 | 107.1  (255.0) | 76 | 34  (21) | 6  (18) | Fenoterol-d6 |
| 4-Hydroxymexiletine | 3.5 | 196.095 | 58.2  (137.1) | 51 | 23  (25) | 10  (8) | Fenoterol-d6 |
| Ipratropium | 4.5 | 332.3 | 166.2  (124.0) | 100 | 36  (45) | 12  (12) | Fenoterol-d6 |
| MDMA | 3.65 | 193.9 | 163.0  (104.9) | 41 | 17  (33) | 10  (6) | Fenoterol-d6 |
| Mecamylamine | 4.26 | 168.179 | 137.1  (80.9) | 66 | 15  (27) | 8  (14) | Fenoterol-d6 |
| Mepenzolate | 3.5 | 341.2 | 130.0  (58.0) | 96 | 39  (85) | 8  (10) | Fenoterol-d6 |
| Meptazinol | 3.55 | 234.469 | 107  (76.9) | 96 | 35  (73) | 20  (6) | Fenoterol-d6 |
| Mescaline | 3.57 | 211.91 | 165  (195) | 46 | 31  (15) | 10  (12) | Fenoterol-d6 |
| 5-Methoxytryptamine | 2.69 | 191.035 | 174.1  (159.1) | 46 | 13  (31) | 10  (10) | Fenoterol-d6 |
| Methylnaltexone | 2.67 | 356.093 | 55.2  (284.1) | 98 | 61  (33) | 10  (18) | Fenoterol-d6 |
| Methylscopolamine | 3.5 | 319.4 | 152.0  (45.1) | 109 | 37  (49) | 10  (8) | Fenoterol-d6 |
| Mexiletine | 5.0 | 180.161 | 58.0  (77.0) | 50 | 21  (52) | 10  (14) | Fenoterol-d6 |
| Milnacipran | 5.6 | 247.174 | 230.2  (100.1) | 51 | 17  (27) | 14  (8) | Fenoterol-d6 |
| MPP+ | 2.68 | 170.016 | 127.1  (154) | 100 | 42  (43) | 8  (10) | Fenoterol-d6 |
| N-Desmethylranitidine | 2.27 | 301.168 | 176  (102.1) | 65 | 21  (41) | 12  (6) | Fenoterol-d6 |
| Neostigmine | 3.54 | 223.193 | 72  (208.1) | 86 | 46  (27) | 14  (14) | Fenoterol-d6 |
| N-Ethyllidocaine | 4.5 | 264.2 | 86.0  (58.0) | 81 | 36  (64) | 16  (11) | Fenoterol-d6 |
| N-Methyltryptamine | 3.68 | 175.105 | 144  (132) | 61 | 17  (15) | 8  (8) | Fenoterol-d6 |
| Nadolol | 3.5 | 310.06 | 254.1  (201.0) | 66 | 23  (31) | 16  (16) | Fenoterol-d6 |
| Ondasetron | 8.3 | 294.1 | 170.1 | 100 | 35 | 15 | Fenoterol-d6 |
| Phenformin | 3.5 | 206.0 | 105.0 | 40 | 35 | 10 | Fenoterol-d6 |
| Pirenzipin | 3.56 | 352.106 | 113.2  (70) | 86 | 29  (63) | 6  (12) | Fenoterol-d6 |
| Practolol | 3.5 | 267.104 | 190.1  (148.1) | 71 | 25  (32) | 12  (10) | Fenoterol-d6 |
| Proguanil | 11.2 | 254.2 | 170.2  (153.1) | 75 | 24  (40) | 10  (10) | Fenoterol-d6 |
| Ractopamine | 4.22 | 302.400 | 107  (91) | 56 | 43  (58) | 6  (16) | Fenoterol-d6 |
| Ritodrine | 3.6 | 288.35 | 270.1  (121.1) | 59 | 19  (31) | 18  (8) | Fenoterol-d6 |
| Rizatriptan | 3.5 | 270.0 | 158.0  (58.0) | 55 | 28  (42) | 11  (11) | Fenoterol-d6 |
| R-Zolmitriptan | 2.67 | 288 | 243  (182) | 75 | 24  (35) | 12  (12) | Fenoterol-d6 |
| Sulpiride | 3.0 | 342.2 | 112.1  (214.2) | 70 | 36  (42) | 15  (15) | Fenoterol-d6 |
| Sumatriptan | 3.57 | 296.2 | 251.2  (58.2) | 50 | 24  (30) | 12  (12) | Fenoterol-d6 |
| S-Zolmitriptan | 2.67 | 288 | 243  (182) | 75 | 24  (35) | 12  (12) | Fenoterol-d6 |
| Tiotropium | 5.7 | 392.081 | 152.1  (170.1) | 81 | 40  (45) | 10  (10) | Fenoterol-d6 |
| Tropisetron | 6.4 | 285.2 | 124.2 | 100 | 30 | 10 | Fenoterol-d6 |
| Trospium | 9.5 | 392.3 | 164.1  (182.2) | 120 | 42  (47) | 12  (12) | Fenoterol-d6 |
| Veralipride | 3.56 | 384.26 | 124.2  (244.1) | 90 | 39  (42) | 8  (14) | Fenoterol-d6 |
| Xamoterol | 3.5 | 340.219 | 253.1  (157.0) | 81 | 22  (33) | 16  (10) | Fenoterol-d6 |
| Xylometazoline | 10.8 | 245.3 | 189.0  (145.0) | 116 | 35  (59) | 12  (10) | Fenoterol-d6 |
| YM155 | 4.02 | 363.13 | 305.3  (93.2) | 100 | 33  (49) | 20  (18) | Fenoterol-d6 |
| **35% organic additive** (64.9% H2O, 0.1 % formic acid, 30.0% acetonitrile, 5.0% methanol) | | | | | | | |
| Almotriptan | 3.3 | 336.219 | 58.2  (201.2) | 71 | 52  (22) | 10  (14) | Bupivacaine |
| Berberine | 3.5 | 336.199 | 320.1  (292.2) | 90 | 40  (40) | 20  (18) | Bupivacaine |
| Bupivacaine | 3.5 | 289.248 | 140.1  (84.2) | 75 | 30  (58) | 8  (16) | - |
| Clidinium | 2.5 | 353.179 | 142.0  (143.1) | 105 | 43  (45) | 8  (10) | Bupivacaine |
| Coptisine | 3.39 | 320.101 | 292.3  (204.2) | 100 | 39  (77) | 18  (12) | Bupivacaine |
| Dehydrocorydaline | 3.82 | 366.132 | 350.2  (322.3) | 101 | 41  (42) | 22  (20) | Bupivacaine |
| Denatonium | 3.1 | 326.500 | 91.01  (86.06) | 71 | 51  (29) | 16  (16) | Bupivacaine |
| Diphenylguanide | 2.51 | 212.1 | 119.1  (77) | 96 | 29  (55) | 22  (4) | Bupivacaine |
| Disopyramide | 3.3 | 340.242 | 239.1  (195.1) | 60 | 25  (41) | 16  (12) | Bupivacaine |
| Dofetilide | 3.36.03 | 442.088 | 198.1  (120.3) | 106 | 39  (65) | 12  (10) | Bupivacaine |
| Epiberberine | 3.36 | 336.027 | 320.2  (292.3) | 101 | 43  (45) | 20  (18) | Bupivacaine |
| Fasudil | 3.55 | 292.063 | 99.2  (70) | 95 | 38  (57) | 8  (12) | Fenoterol-d6 |
| Fenfluramine | 3.4 | 232.122 | 159.2  (109.1) | 66 | 29  (59) | 14  (6) | Bupivacaine |
| Gabexate | 4.23 | 322.083 | 96.1  (121.1) | 106 | 34  (32) | 18  (8) | Bupivacaine |
| Glycopyrrolate | 3.61 | 318.313 | 116.1  (58.3) | 90 | 41  (77) | 8  (10) | Bupivacaine |
| Harmaline | 3.3 | 214.8 | 174.1  (200.1) | 96 | 31  (31) | 12  (12) | Bupivacaine |
| Methaiodobenzylguanidine | 3.35 | 276.09 | 217  (90) | 76 | 29  (54) | 14  (16) | Bupivacaine |
| Methylene blue | 3.8 | 284.0 | 268.0  (251.1) | 101 | 47  (67) | 8  (16) | Bupivacaine |
| Minoxidil | 3.3 | 210.15 | 193.2  (164.1) | 70 | 21  (32) | 12  (12) | Bupivacaine |
| Olodaterol | 2.5 | 387.199 | 163.1  (207) | 70 | 27  (28) | 10  (14) | Bupivacaine |
| Oxymetazoline | 3.8 | 261.173 | 205.2  (135.2) | 111 | 35  (45) | 12  (8) | Bupivacaine |
| Oxyphenonium | 4.0 | 348.237 | 73.0  (132.2) | 96 | 47  (41) | 14  (8) | Bupivacaine |
| Palmatin | 3.58 | 352.156 | 336.3  (308.2) | 90 | 39  (39)  20 | 20  (20) | Bupivacaine |
| Propanthelinbromid | 4.96 | 368.125 | 181.1  (100.2) | 75 | 40  (32) | 12  (18) | Bupivacaine |
| (R)-Aclidinium | 4.3 | 484.185 | 262.2  (140.2) | 110 | 45  (72) | 18  (10) | Bupivacaine |
| Rucaparib | 3.3 | 324.2 | 293.2  (236.0) | 46 | 13  (47) | 18  (14) | Bupivacaine |
| (S)-Aclidinium | 4.3 | 484.185 | 262.2  (140.2) | 110 | 45  (72) | 18  (10) | Bupivacaine |
| Tamsulosin | 3.4 | 409.227 | 228.1  (271.1) | 91 | 33  (27) | 14  (18) | Bupivacaine |
| Trospium | 3.5 | 392.3 | 164.1  (182.2) | 120 | 42  (47) | 12  (12) | Bupivacaine |
| **50% organic additive** (49.9% H2O, 0.1 % formic acid, 42.9% acetonitrile, 7.1% methanol) | | | | | | | |
| Propranolol | 4.3 | 372.232 | 71.9  (70.0) | 90 | 47  (75) | 14  (6) | - |
| Umeclidinium | 3.1 | 428.258 | 96.2  (91.0) | 120 | 68  (77) | 18  (18) | Propranolol |
|  |  |  |  |  |  |  |  |
